# Management of Motion and Anatomical Variations in Charged Particle Therapy: Past, Present, and Into the Future

**DOI:** 10.3389/fonc.2022.806153

**Published:** 2022-03-09

**Authors:** Julia M. Pakela, Antje Knopf, Lei Dong, Antoni Rucinski, Wei Zou

**Affiliations:** ^1^ Department of Radiation Oncology, University of Pennsylvania, Philadelphia, PA, United States; ^2^ Department of Radiation Oncology, University Medical Center Groningen, University of Groningen, Groningen, Netherlands; ^3^ Department I of Internal Medicine, Center for Integrated Oncology Cologne, University Hospital of Cologne, Cologne, Germany; ^4^ Institute of Nuclear Physics, Polish Academy of Sciences, Krakow, Poland

**Keywords:** motion management, 4DRT, pencil beam scanning (PBS), particle therapy, proton therapy

## Abstract

The major aim of radiation therapy is to provide curative or palliative treatment to cancerous malignancies while minimizing damage to healthy tissues. Charged particle radiotherapy utilizing carbon ions or protons is uniquely suited for this task due to its ability to achieve highly conformal dose distributions around the tumor volume. For these treatment modalities, uncertainties in the localization of patient anatomy due to inter- and intra-fractional motion present a heightened risk of undesired dose delivery. A diverse range of mitigation strategies have been developed and clinically implemented in various disease sites to monitor and correct for patient motion, but much work remains. This review provides an overview of current clinical practices for inter and intra-fractional motion management in charged particle therapy, including motion control, current imaging and motion tracking modalities, as well as treatment planning and delivery techniques. We also cover progress to date on emerging technologies including particle-based radiography imaging, novel treatment delivery methods such as tumor tracking and FLASH, and artificial intelligence and discuss their potential impact towards improving or increasing the challenge of motion mitigation in charged particle therapy.

## Introduction

The use of charged particles for radiation therapy (RT) represents a valuable treatment paradigm because their unique dose deposition properties, including maximum dose deposition at the Bragg peak and rapid distal falloff, allow for dose to be conformed tightly around the tumor while sparing normal tissues (1–6). However, these advantageous properties present a challenge in the presence of motion, because the same steep dose gradients which provide the benefit of lower integral dose in surrounding tissues are vulnerable to even small displacements in the patient geometry. In addition, because their range is dependent on tissues along the beam path, charged particles traveling through heterogenous tissues (such as in lung cancer) also suffer from dose deviations due to motion-induced range uncertainties (7).

Proton therapy is by far the most widely implemented form of charged particle therapy, with forty-one proton centers operating in the US and ninety-nine centers operating worldwide (8). Initial proton therapy systems were based on passive scattering, in which a scattered beam is passed through a range-modulating wheel [to determine the width of the spread-out Bragg peak (SOBP)] and a custom collimator and compensator (to laterally shape the beam and match the distal edge of the SOBP to that of the patient target). However, this scattering method is limited in its ability to conform dose to the proximal boundary of the target (9). Over time, a pencil beam scanning (PBS) method was developed which exhibited improved dose conformation around the target in comparison to passive scattering methods; dosimetric benefits of proton PBS have been verified across a diverse range of disease sites, including brain, esophageal, oropharyngeal, breast, and liver cancers (3, 6, 10–14). Proton PBS is performed by scanning a monoenergetic pencil beam over a grid of positions and repeating for multiple beam energies to create a SOBP with a varying span along the lateral direction. Treatments using PBS delivery utilize intensity modulation of the pencil beam spots and are optimized using one of two planning techniques. Single field uniform dose (SFUD) planning refers to a planning technique in which pencil beam weights are optimized independently for each field according to the planning objectives, resulting in treatment plans where each field contributes similar tumor coverage. Multi-field optimization (MFO) is a planning technique in which the pencil beam weights of every field are optimized simultaneously. Of these two planning methods, SFUD is more robust against motion because it only requires the consistency of anatomy during the treatment delivery of its own field. MFO requires the integrity of anatomy during the entire treatment session from all treatment fields; therefore, the potential impact of motion is more significant. PBS-based proton therapy is often referred to as intensity-modulated proton therapy (IMPT), which is created by simultaneously optimizing the beam intensity and energy for each spot/delivery unit. The addition to range uncertainties and sensitivity due to steep dose gradients at the distal end of the target, proton PBS presents an additional challenge for precise dose delivery under conditions of motion due to interplay effects between the scanning beam and intra-fractional motion in the patient anatomy (5, 15). The interplay of the proton and ion pencil beam scanning and organ motion has been shown to impact the target dose coverage in multiple disease sites (16–19). Recent lung cancer simulation studies found that interplay effects combined with small spot sizes (~3mm) resulted in a loss of 2-year local control of up to 18.5% ± 25.2% compared with the static cases for single fraction delivery; for multi-fractionation treatment significant loss of local control was observed only for large motion amplitudes (>30mm) (16). For esophageal cancer, a recent planning study found PBS proton therapy plans using posterior beams to be more robust against underdosage (V95%_CTV_ <97%) in comparison with IMRT, but interplay effects had an increasingly negative impact on the dose distribution as the number of simulated fractions was decreased below 10 (17). As the beam delivery time structures and intensities from various charged particle accelerators such as synchrotron or cyclotron are different (20), they can result in different interplay effects (21).

As of 2021, there currently exist twelve operating carbon-ion treatment centers with five more under construction (8, 22). Similar to protons, carbon and other heavier ions such as helium exhibit highly localized dose deposition at the Bragg peak. An additional advantage of treating with carbon ions (over photons and protons) is that they exhibit steeper lateral penumbra and higher linear energy transfer (LET), leading to increased relative biological effectiveness (RBE) (23). Like protons, carbon ions can be delivered either *via* passive scattering or pencil beam scanning; however, largely due to cost the majority of centers perform only fixed beam delivery, with only two capable of a full range of treatment angles through rotating gantry (24). A challenge of treating with carbon and other heavy ions is that the increased sharpness of the lateral penumbra in combination with the Bragg peak results in heightened dose uncertainties in the presence of motion. In addition, because carbon ions exhibit a distal tail of dose beyond the Bragg peak due to nuclear fragmentation, the potential for dose uncertainties distal to the target volume are of greater concern (24). Additionally, for carbon and heavier ions, RBE is nonlinear with respect to the absorbed dose level, particle energy, and atomic number (25), thus while it is typical to consider a constant RBE for proton radiotherapy (26), when considering the carbon ion interplay effects, the changing RBE along the beam path should be taken into consideration.

Motion management refers to techniques in RT which seek to mitigate the effects of inter- and intra-fractional motion. Causes of inter-fractional motion include weight loss, tumor shrinkage, and organ deformations such as those caused by changes in the volume of the rectum and bladder (27, 28). Inter-fractional motion occurs between fractions, typically separated by hours or days, and thus if correctly identified can be corrected with an established adaptive planning process. Intra-fractional motion is the result of respiration and to a lesser extent cardiac motion and peristalsis. Because intra-fractional motion occurs during treatment on a timescale of minutes to seconds, it represents a greater challenge. Imaging and treatment planning modalities which take into consideration time in addition to the 3-dimensional space of the patient are commonly referred to as 4D (e.g., 4DCT and 4DRT).

Motion management can generally be categorized into one of two strategies: 1) prevent or reduce anatomical motion, or 2) adapt treatment to motion during planning or delivery. The success of these strategies depends on access to imaging techniques which allow motion and/or dose deposition to be faithfully monitored and quantified. While much work has been done to address challenges in motion related to charged particle therapy, currently there is limited standardization of treatment protocols, with many centers developing their own unique standard of procedures and best practices (29). For example, in the case of proton therapy for thoracic cases, the Particle Therapy Co-Operative Group (PTCOG) Thoracic and Lymphoma Subcommittee has begun the work to define a set of guidelines for best practices, but stress that it is still necessary for institutions to determine their own protocols, due to variations in technology across institutions as well as lack of commercialization for many solutions (9). Recommendations for control at other treatment sites are scarce.

This review seeks to provide an overview of current clinical practices for intra- and inter-fractional motion management in charged particle therapy as well as progress to date on emerging technologies that have potential to be used for or impact motion management. The remainder of this review is organized as follows: Section 2 provides an overview of the current state of the art in motion management implemented in clinical charged particle therapy, including motion reduction techniques at the major anatomical sites, imaging techniques for monitoring intra- and inter-fractional motion, as well as treatment planning, delivery, and plan evaluation mitigation strategies; Section 3 discusses promising ongoing research and emerging technologies in charged particle therapy which will either directly benefit or necessitate further innovations in motion management.

## Clinical Implementation: the Current State of the Art

### Motion Control Techniques at Simulation and Delivery

Motion control techniques consist of methods which seek to reduce patient motion and ensure consistent geometrical orientation during and between fractions.

#### Thorax and Upper Abdomen

In the thorax and upper abdomen, respiration is the most significant cause of intra-fractional anatomical variations. In thoracic tumors, motion control methods for proton therapy are recommended when a patient’s target motion amplitude exceeds a pre-determined threshold (established per institution based on target location and treatment parameters) (9). Breath-hold is a standard technique for minimizing respiration-based movement during treatment delivery to the thorax and abdomen. Breath-hold involves patients temporarily suspending respiration at specific, reproducible lung volumes. Breath-hold techniques can be classified as *voluntary*, in which the patient is in control of their breathing (30), or *active*, in which the patient’s airflow is temporarily blocked by a valve (31). A widely used commercial voluntary breath-hold system is the SDX Respiratory Gating System (DYN’R Medical Systems, Toulouse, France). The SDX system monitors the inspiration and expiration volume of a patient in real-time and provides visual display of the breathing-trace through patient-worn goggles. This allows the patient to perform consistently reproducible breath-holds at a specific volume for a specific duration of time. Typically, for thoracic cases, deep inspiration breath-hold (DIBH) is preferrable (holding in a deep breath at an individually determined volume threshold) because this increases the distance between the tumor and organs at risk. DIBH proton treatments to Hodgkin lymphoma and left breast cancer have shown significant reduction in lung and heart dose compared with the photon plans (32–34). High frequency percussive ventilation (HFPV) is an example of an active breath hold technique. In HFPV, the patient receives high frequency pressured pulses of air, causing suppression of respiratory motion and allowing for longer breath-holds. A drawback of HFPV is the requirement of anesthesia support, which may not be always available in radiation oncology. A recent study has also reported on the use of an enhanced deep inspiration breath hold (eDIBH), which involves passive, patient-controlled breath holds aided by preoxygenation, hyperventilation, and patient coaching to increase breath-hold times. eDIBH was found to reduce variability in lung volume and position in comparison with HFPV (35). Not all patients are candidates for breath-hold techniques. Patients with impaired respiratory function typical of advanced lung cancer may be physically unable to tolerate breath holds, while younger patients may be unable to follow instructions for voluntary breath-hold. Another limitation of breath-hold based motion management is it cannot account for inter-fractional anatomical changes, such as tumor shrinkage and lung density changes which can result in changes to the water equivalent path length (36).

Abdominal compression (AC) belts are a relatively inexpensive and easy-to-implement option for minimizing intra-fractional motion from respiration. For charged particle therapy, thin belts made of homogenous materials are preferred to ensure that the belt has a minimal and reproducible impact on the charged particle range (37). For PBS proton therapy, the use of AC has been shown to be valuable for reducing moderate to large motion during treatment of liver tumors (37, 38). Additionally, AC in combination with layered rescanning and respiratory gating has been clinically adopted for motion mitigation in carbon ion therapy (39). A limitation of compression belts is that the degree of compression applied is limited by individual patient tolerances, with some patients are unable to tolerate abdominal compression during treatment. The compression belt typically has fiducial markers and pressure meter to ensure consistent positioning of the belt during treatment. Nonetheless, it is recommended to avoid having particle beam passing through the device to avoid potential water equivalent thickness (WET) variations in the setup (38).

#### Lower Abdomen and Pelvic Regions

For the lower abdomen and pelvic regions, positioning techniques focus on reducing motion caused by volume changes in the rectum and bladder. Consistency of the position of bladder and rectal volume across and during treatment can be improved through hydration and voiding schedules (40, 41). Rectal balloons are used to immobilize the prostate and maintain the rectum at a consistent volume during simulation and treatment, reducing uncertainties from variable rectal filling and gas and allowing for smaller target margins and greater sparing of the rectum during external beam radiotherapy (42). For charged particle therapy, rectal balloons are inflated with water instead of air to maintain a consistent water equivalent pathlength (41); this removal of the rectal air cavity likely reduces the dosimetric benefit to the anterior rectal wall which is seen with the air-based inflation used in photon therapy (42, 43). Recently, a rectal biodegradable hydrogel that is implanted between the prostate gland and the rectum was implemented in the clinic. For proton radiotherapy, the use of hydrogel provided improved rectal sparing and decreased late gastrointestinal toxicity compared with the use of a rectal balloon, suggesting that motion due to moderate rectal filling does not require intervention if adequate separation (8-13mm in this study) between prostate and rectum is achieved (44).

### Imaging at Simulation and Delivery

Imaging technologies play a pivotal role in motion management for radiation therapy, allowing for the contouring of structures for treatment planning and providing a means to assess and monitor organ motion, either directly by imaging the motion of organs or *via* indirectly by imaging markers placed in tissue, e.g., fiducial markers. Computed tomography (CT) images are the gold-standard modality for treatment planning; however, CT images acquired during free breathing can suffer from motion artifacts, leading to uncertainty in the true delineation of boundaries between tumor, normal tissues, and organs at risk (45). For this reason, 4D computed tomography (4DCT) images, in which a series of CT images are acquired during different phases of the breathing cycle, have become a standard in treatment planning for thoracic and abdominal regions. One limitation of 4DCT is that irregularities in patient breathing during image acquisition leads to inherent uncertainties in how faithfully the final 4D images represent the patient’s anatomy during respiration. Patients can also exhibit variations in their breathing cycle during day-to-day treatment; the intra-fractional motion patterns from respiration can themselves be subject to inter-fractional variations. For proton PBS therapy, weekly offline CT assessment are recommended for patients expected to experience inter-fractional changes (9). Because not all commercial treatment planning software supports multi-image planning, information from the 4DCT can be utilized for treatment planning by transforming the series of images into a single “representative” view of the patient, such as average intensity projection (AIP), maximum intensity projection (MIP) and mid-ventilation (MidV) (46).

Simulation for proton therapy treatment planning often involves fusion with tissue function-related positron emission tomography (PET) and magnetic resonance imaging (MRI) for target delineation. 4DPET provides information on tumor motion in addition to the metabolic information about the tumor structure and has been shown to be a valuable tool for target delineation in lung cancers (47). 4DMRI provides high contrast of soft tissues and has shown promise for motion-robust target delineation in both abdominal and lung cancers (48–50). A recent study further used 4DMRI and 3DCT to generate synthetic 4DCT for 4D dose calculation and interplay effect evaluation in pancreatic cancer (51).

Conventional cone-beam CT (CBCT) was first implemented for proton therapy for patient setup in 2012 (52). Daily CBCT adds valuable 3D anatomy information and soft tissue contrast in patient setup alignment compared to 2D kV imaging. It can also be used to identify inter-fractional tumor regression, weight loss, organ filling or atelectasis. Efforts have been made to enable CBCT for online adaptive planning for proton therapy (53–56), but it suffers from reduced image quality in comparison to CT due to restricted field of view, lower soft-tissue contrast, and inequivalent relation between CBCT voxel values and Hounsfield units. CT-on-Rails has also been investigated as an alternative to daily CBCT imaging for daily adaptive radiotherapy due to its ability to provide higher quality images at “near-treatment-position”, though widespread adaption of CT-on-Rails is limited due to increased cost and larger footprint (57). Interested readers can find an in-depth discussion on radiological image guidance in particle therapy in the recent review by Landry and Hua (58).

Internal tumor motion during delivery can be monitored through implanted markers, such as electromagnetic sensors (59–61) and fiducial markers (62), with externally placed detectors. A benefit of EM systems or on-board imagers is that they do not require line-of-site and can thus also capture signal from internally placed markers. Tang et al. have used electromagnetic transponders to assess the intra-fractional prostate motion during PBS SFUD delivery (63). A major limitation of electromagnetic systems is that they are sensitive to distortions of the generated electromagnetic signal; a recent study found EM tracking systems unable to provide clinically useful measurements during proton therapy in the presence of gantry motion or when too close to a CT scanner (61). In addition, caution should be taken in the selection of the implanted markers for proton therapy to avoid image artifacts and dose disturbance (64).

Fluoroscopy with implanted markers can produce 2D images with high spatial and temporal resolution. Researchers at Hokkaido University in Japan have pioneered the clinical implementation of a gated proton PBS treatment system that utilizes fluoroscopy for real time tumor tracking through an internally placed gold fiducial marker (65–67). Aside from requiring an invasive placement, a potential risk is that the fiducial marker could migrate from its original placement during the course of treatment (68).

Another strategy for assessing tumor motion is to measure the patient’s surface motion, which can then be used as a surrogate to infer the position and movement of the internal target (69). Surface imaging uses cameras/projectors mounted in the treatment room to capture pseudo-random light patterns projected on the patient surface to derive the patient surface change. It is non-ionizing and can monitor a large surface constantly from a distance; thus in addition correcting for inter-fractional motion during daily positioning, surface imaging is an attractive option for monitoring intra-fractional motion (70). A reported challenge of surface imaging in a clinical proton setting is that the layout of the therapy room can lead to restricted field of view or occlusion of the surface cameras at treatment gantry angle (71). Clinical applications of surface imaging for daily positioning of breast cancer proton therapy patients have been reported and found to achieve similar dosimetric accuracy to daily CBCT (70–72); however it was noted that surface imaging alone can be insufficient to detect internal shifts of breast implants (71). Further surface imaging studies have noted limited correspondence between measured surface motion and internal target motion (73–75). Nevertheless, a recent technological assessment showed that optical tracking has better potential than electromagnetic tracking of embedded fiducial markers for use in PBS proton therapy (61).

### Treatment Planning and Delivery

Many different strategies exist to account for patient motion during the treatment planning process or delivery itself. Mitigation during treatment planning and delivery is characterized by an approach of working with or around existing patient motion. These strategies rely on additional information input from imaging, either during simulation (in the case of treatment planning) or in real time during treatment delivery.

#### Motion-Encompassing Margins

One of the most widely used planning methods to account for expected patient motion in charged particle therapy is to delineate an additional margin around the tumor target, commonly denoted the internal target volume (ITV), which is defined as the overlay of boundaries for all (or a select subset of) CTV positions collected during 4DCT (76). While the use of ITVs alone is not considered adequate for motion management in PBS proton therapy due to range uncertainties and interplay effects, ITVs are recommended for use in combination with other motion mitigation strategies to account for any residual motion uncertainties (77). For example, for patients being treated with breath-hold, several breath-hold CT scans are performed during simulation to create an ITV that encompasses the variability of the breath-hold position for a particular patient. ITV structures can also be used in conjunction with compression belts under conditions of free breathing to account for residual motion. ITVs can be constructed using different methods based on 4DCT images, including Max/Min inhale-based, MIP-based, and MidV-based. In addition, the concept of using density overrides within and around the ITV has been shown in recent simulation studies to improve dose coverage when the target moves through regions of highly variable density (78, 79). Once an appropriate ITV has been determined, additional population-based margins reflecting setup and beam-specific range uncertainties are applied to generate a planning target for each beam (80).

#### Repainting

A unique challenge for particle therapy in the face of intra-fractional motion is the additional dose uncertainty caused by interplay effects between patient anatomical motion and the delivery sequence of the scanning beam. A mitigation method is to use repainting, which refers to a treatment delivery method applied for PBS by rescanning over the same spot positions multiple times (using an appropriately lower fraction of monitor units defined by the total number of repetitions) (81, 82). Studies on repainting for PBS proton therapy have found that this method improves dose homogeneity; however, interplay effects were found to be more pronounced for small tumor sizes, motion greater than 10mm, and small spot sizes (83, 84). Another recent study demonstrated that interplay effects due to motion could be further reduced using a novel repainting strategy in which the repaintings for a given energy layer (and by extension the repaintings for each spot position) are spread out evenly over the entire breathing cycle (85). This was accomplished by designing a flexible delivery schedule in which the number of times a given spot is repainted as a function of its assigned MU and the time it takes for the scanned beam to reach the given spot from its previous position, such that: 1) each spot is repainted as many times as possible, 2) repaintings for each spot are spread evenly over the breathing cycle, and 3) the total time to deliver a given energy layer is constricted to the length of the breathing cycle. One challenge for the repainting strategy is that it may encounter the minimum MU requirements because it requires the delivery of a smaller amount of dose multiple times. Sometimes it may only be possible to repaint the distal layers because they usually receive more dose.

#### Beam Angle Selection and Spot Size

In treatment planning, beam angle selection can help to minimize dose deviations by selecting beam orientations that are robust to motion. Proton range is sensitive to tissue variations along the beam path. Movement can cause tissue variation uncertainties that lead to dose deposition uncertainties. Thus, in selecting beam angles for proton therapy, it is advantageous to avoid large tissue density gradients in the beam path and to try to keep the beam angle parallel to the dominant direction of tumor motion (9). It is also important to avoid beam orientations such that the distal fall-off region of the proton range proximally borders an OAR, as range uncertainties could lead to unintended overdosing OAR or underdosing the CTV. Specific protocols for the selection of beam angles have been found to vary across facilities, but nevertheless follow these guidelines discussed above (76).

The pencil beam spot size, defined as the full width at half-maximum of the beam spot in air, has been identified as an additional beam parameter that impacts dosimetric deviations in PBS proton therapy (16, 63, 76). Specifically, larger spot sizes (~13 mm) have been shown to reduce the interplay effect compared with smaller spot sizes (~3mm) (16).

#### Respiratory Gating

Respiratory gating is a dynamic treatment delivery method used to ensure precise dose deposition in the presence of respiratory motion. In this technique, the treatment beam is turned on only when the patient is in a specific phase of their respiratory cycle. Gating methods can be performed in conjunction with breath-hold techniques or during free breathing; clinical use for charged particles has been described for several disease sites, including the lung, breast, and liver (86–89). Implementation of gating relies on the ability to accurately monitor patient motion during treatment delivery and on the ability for patients to perform reproducible breath holds or reproducible free-breathing cycles. Motion monitoring for charged particle gating is typically accomplished *via* external surface imaging (90). For PBS proton therapy, a gating approach to treatment delivery in conjunction with real-time fluoroscopy imaging has been implemented and shown *via* simulation studies to be capable of significantly reducing mean liver dose while providing better target coverage in comparison with a free-breathing approach (66, 91). A limitation of beam gating for PBS-based therapies is that they can still suffer from interplay effects due to residual motion during the selected gating window; recent simulation studies suggest this can be mitigated through the use of laterally and longitudinally overlapping pencil beams or phase-controlled rescanning (92, 93). Another reported challenge to the clinical use of gating for PBS is balancing the gating window width with the duty cycle (ratio of beam-on time to overall treatment time). While a narrower window width provides improved motion mitigation, it can also lead to unacceptably long treatment times. A recent simulation study for PBS-based gated therapy of liver tumors found duty cycle dropped by as low as 10% when a small window width (3mm) was used for a gating-only approach (94). Gating in combination with repainting has been shown to be more efficient in motion mitigation than repainting alone (94, 95). Another challenge of implementing gating for proton therapy is that proton systems are subject to latency in beam control, meaning once a gating signal is detected from the patient, the treatment machine requires a delay to turn-on or turn-off the beam. This delay can sometimes be too long (150-200 ms in a 3-4 s breathing cycle) to perform reliable cycle-to-cycle beam delivery. For this reason, respiratory gating in particle therapy is usually performed in conjunction with breath-hold techniques, which provide a longer treatment window such that the beam’s gate-lag time is insignificant.

#### 4D and Robust Optimization

In radiation therapy, robust optimization (RO) can be used during the treatment planning process to design plans which are robust to the uncertainties associated with treatment delivery, including setup errors and patient motion. Traditionally for photons, such uncertainties in treatment planning have been accounted for by using an expanded margin around the CTV, called the planning target volume (PTV). In proton therapy, the additional presence of range uncertainties and, in the case of PBS, uncertainties from the interplay effect render the use of traditional margin-based solutions insufficient.

3DRO refers to a class of robust optimization techniques which account for patient setup and range uncertainties during treatment plan optimization. Implementations of 3DRO include probabilistic treatment planning, scenario-wise mini-max RO, and voxel-wise worst-case RO. In probabilistic optimization, setup and range uncertainty scenarios are randomly sampled from an assumed probability distribution to produce a large set of potential dose distributions (96, 97). This method is computationally expensive and requires prior knowledge of the probability distributions of the treatment conditions. Scenario-wise mini-max and voxel-wise worst-case RO evade these challenges by performing plan optimization based on either a smaller set of worst-case scenarios or a single worst-possible dose distribution (98–100). 4DRO can be considered an extension of 3DRO in that it also considers intra-fractional variations caused by organ motion such as respiration. Recent simulation studies have shown promising benefits of 4DRO for scanning proton therapy, including improved target coverage and improved robustness against interplay effects (100–102). In a study by Ge et al. comparing the performance between 3DRO and an in-house 4DRO system on a set of 10 lung cancer patients treated with IMPT, the 4DRO system was found to outperform both 3DRO and traditional PTV-based optimization methods in terms of dose homogeneity, CTV target coverage, and dose robustness considering setup uncertainties and patient motion (100). Another recent study published by Mastella et al. looked at the incorporation of gating with 4DRO for 20 lung cancer patients found that this 4DRO optimized gating approach resulted in significant reduction to lung dose while maintaining target coverage (102).

Studies have been performed for carbon ion 4D dose reconstruction and optimization. Reconstruction of the delivered 4D RBE dose needs to consider the tumor motion and temporal structure of the beam delivery (103). Eley et al. (104) worked on a 4D optimization approach for scanned beam tracking to reduce the dose to organs near the moving target. Graeff et al. (105) proposed a 4D optimization scheme that divides the target into sectors and to each of the sector with a delivers dedicated raster field corresponding to individual motion phases. Expanded carbon ion 4D-IMPT including the robust non-linear RBE-weighted optimization was also included (106, 107). For clinical application, it is essential to consider both the 4D treatment planning systems and 4D treatment control systems (108). 4DRO is currently commercially available through the RayStation treatment planning system (RaySearch Laboratories, Stockholm, Sweden) (109).

#### Plan Evaluation

Due to the interplay effect in proton PBS delivery, special considerations should be taken in evaluating PBS proton plans to ensure that the intended dose distribution can be delivered faithfully under conditions of anatomical motion. The guidelines provided by the Particle Therapy Co-Operative Group (PTCOG) Thoracic and Lymphoma Subcommittee recommended the use of a 4D phantom to evaluate motion interplay effects and the effectiveness of planned motion mitigation techniques, though at this time such a protocol is admittedly out of reach for most centers due to the expense of 4D phantoms (9), which have only recently become commercially available. In the same publication, the importance of continuous plan evaluation (through regular repeat 4DCT scans) to assess the need for plan adaption is also emphasized. Meijers et al. reported on a 4D dose reconstruction method which utilizes weekly repeated 4DCT scans in combination with treatment log files and breathing pattern records from each fraction (110, 111). This dose reconstruction method can be used to calculate dose accumulation on a per-fraction basis, allowing the clinical team to monitor motion-based dose degradation over the course of treatment and to help to trigger adaption when necessary. Recent studies have also reported on the implementation of tools designed for evaluation of PBS proton plans for moving targets. For example, Riberio et al. reported on the development and application of a plan evaluation tool, 4D robust evaluation method (4DREM), which considers both setup errors as well as respiratory motion and the interplay effects (112), while Korevaar et al. developed a scenario-based plan evaluation method which allows for comparison between photon and proton plans (113). In another hand, fast 4D dose calculation with GPU-accelerated dose-engines enable potential clinical use and can be integrated into the optimization process (114–116). Another consideration for 4D dose calculation is that the temporal resolution used can impact the calculated final accumulated dose distribution, a recent simulation study found that the use of finer temporal resolutions for 4D dose calculations can help to reduce the over-estimation of interplay effects for hypo-fractionated treatments (19).

## Future Technologies

### Advanced Imaging Techniques

A major directive in motion management is to develop methods that allow for high quality *in vivo* observation of internal anatomic structures during treatment fractions. 4D-CBCT has been recognized as a promising emerging technology in identifying intra-fractional motion at the time of treatment and has been investigated for photon therapy (117, 118). This technology potentially reduces the motion artifacts associated with 3D-CBCT and can be used for tumor motion verification when comparing with the planning 4DCT (119). Currently, there is a trade-off between image acquisition time and image quality, making it challenging for fast and reliable adaptive tumor target delineation and motion analysis with 4D-CBCT. Research investigations of image processing methods to improve 4D-CBCT image quality with reduced scan times are underway (120–123), and the commercial development of a dual-imaging proton treatment system with 4D-CBCT capability has been announced by Hitachi, with approval of the Japan’s Pharmaceuticals and Medical Device Agency (PMDA) granted in 2020 (124).

Other imaging approaches provide promising alternatives for *in vivo* monitoring of anatomical changes and assessment of range uncertainties. One such developing imaging method for charged particle therapy is ion-based radiography, such as proton radiography (PR). PR involves measuring the residual energy of high energy protons after they have passed through the patient to construct a 2D image where each pixel represents the WET of the patient along the beam path at that position. PR has been proposed as a tool for quality control through *in vivo* proton range verification, as an aid for pre-treatment patient positioning, and even as a means to guide online plan adaptations (125, 126). The characterization of the WET accuracy, noise, and spatial resolution of a commercially developed prototype for clinical PR were reported (127, 128). Another PR system which measures individual proton depth-dose curves *via* a multi-layer ionization chamber was introduced and used to measure range uncertainties in proton pencil beams in a 4D porcine lung model; the findings supported the use of 3% range uncertainties for robust treatment planning in thoracic regions (129, 130). Proton-based fluoroscopy has also been proposed, and was investigated for range verification and monitoring of inter- and intra-fractional motion in lung tumors in a recent simulation study (131). Helium and carbon ion-based radiography systems have also been investigated in simulation and experimental studies, with carbon ions achieving the highest image quality at the cost of increased dose (132–134). A benefit of ion-based radiography is that it requires less dose in comparison with conventional x-ray radiography due to low fluence requirements and the Bragg peak not contributing to patient dose. A limitation of ion-based radiography is that particles must be able to fully penetrate the patient to acquire an image. Current clinical therapy systems can achieve a penetration depth of ~30cm, which may be insufficient for large patients for certain beam directions.

MRI-guided radiotherapy is an exciting imaging strategy for *in vivo* motion management during treatment because in addition to being non-ionizing, it provides excellent soft tissue contrast, high temporal resolution, and is capable of functional imaging (135, 136). While it has already been implemented for photon radiotherapy *via* the MR-linac, the application to charged particles is currently challenged by the need to mitigate the deflection of the treatment beam by the beamline magnetic field. Modelling on MR-integrated proton therapy for liver cancer has shown clear dosimetric benefits as well as significant reduction in normal tissue complication probability in comparison with other imaging modalities, including offline MR-guided proton therapy and MR-linac (137). Recent studies have made progress in quantifying the impact of the magnetic field on detectors for proton dosimetry and demonstrating the technical feasibility of low-field MR guidance on phantoms in a static research beam line (138, 139). Additional studies have investigated methods for calculating stopping power ratios from MRI to generate an MRI-based “synthetic” CT image for treatment planning and range verification (140). The first clinical MRI-proton system is scheduled to be built in 2022 at the OncoRay – National Center for Radiation Research in Oncology in Dresden, Germany (141).

Motion changes the ion beam path and alters the beam range. *In vivo* ion beam range can be potentially monitored by secondary particles generated during nuclear reactions of the ion beams with tissue (142). One set of the nuclear processes generates position emitters which annihilates into 511 keV photon pairs. Several in-beam PET designs were proposed to detect the proton range (143, 144). Its potential to assess the proton and carbon ion beam ranges was studied (145, 146). However, the limited PET spatial resolution and the biological washout of the nuclear activation confounds the analysis of the range from the PET images. Nonetheless, reconstructed time-resolved activities indicates its potential for online range verification during treatment (147).

Prompt gamma (PG) imaging is a technology that allows for *in vivo* proton range verification (on a spot-by-spot basis) during treatment delivery through the measurement of gamma rays created from nuclear interactions with protons passing through tissue. The *in vivo* human applications of prompt gamma with passive scattering and PBS proton beams were reported (148, 149). One challenge of implementing PG imaging is that the number of photons needed to create a reliable signal (~10^8^) exceeds the number of photons generated in most pencil beams for RT treatment. Potential solutions to this challenge include re-optimizing the treatment plan such that several of the pencil beams are boosted to reach this counting threshold, or aggregating the statistics from multiple neighbour pencil beams (at the cost of reduced resolution) (150). A recent simulation study by Tian et al. demonstrated under conditions of inter-fractional motion that a selective spot boosting method achieved tight <0.8 mm PG-dose correlation. This study further supports its proposed use for proton range monitoring (151).

Detection of the secondary charged particle emission in light-ions such as carbon ion beams was also proposed for beam range monitoring purposes (152); the capability of using charged secondary emission profiles to track ion beam range, spot position, and internal motion has been demonstrated in recent simulation studies (153, 154). It is realized by back-tracking and reconstructing the profile of the secondary emissions (155). The use of charged particles to detect lateral pencil beam position to a resolution of millimeters was reported recently in anthropomorphic head and neck phantom using a carbon ion beam (156). The INnovative Solutions for In-beam Dosimetry in hadronthErapy (INSIDE) collaboration has recently reported on the clinical trial for a carbon ion beam-range monitor and demonstrated the potential of this system to detect inter-fractional beam range variations which could be used to trigger re-simulation and adaptive replanning (157).

Another emerging technology for *in vivo* range verification is protoacoustics. Protoacoustics works by measuring the thermoacoustic pressure waves generated by the energy deposition of therapeutic protons in tissue. Like prompt gamma, protoacoustics is an attractive option for range verification in that it is derives its signal from the treatment beam itself, essentially utilizing free information from the patient’s internal anatomy without the need for additional dose. In addition, a protoacoustic system can be implemented at low cost compared with other methods (158). Currently, the primary challenge in implementing protoacoustics as a mainstream technology is to develop methods for rapid and accurate translation of the acoustic pressure signal in heterogenous tissues into meaningful information about proton range, which is an active area of research (159, 160). Further work has also been done to characterize the dependencies of the protoacoustic signal on the proton pulse shape (161).

### Novel Delivery Methods

Tumor tracking or “beam tracking” in radiotherapy refers to a delivery method in which the tumor position is actively tracked, and the treatment beam is modified in real-time to correct for intra-fractional motion. It has been described by previous reviews as one of the most promising but also the most technically challenging motion mitigation strategy for charged particles (162–164). Although tumor tracking has been achieved in the clinic for photon therapy, clinical implementation has yet to be realized for scanned ion beams due to additional technical challenges presented by charged particle treatment regimes. Unlike photon therapy in which tumor tracking can be achieved in the beam’s eye view, tumor tracking with scanned ion beams requires 3D information as well as the ion beam delivery system’s ability to change beam energy rapidly. Specifically, both the beam position and beam energy need to be rapidly modified to account for changes in the location of the tumor as well as the changes in the beam range caused by variations in depth and/or tissue composition in the beam path. Simulation studies have evaluated the robustness of tumor tracking systems for both carbon ion and proton scanned beams (165, 166). Ultrasound-guided tracking systems have also been explored (167, 168). Efforts have also been made to explore a motion-synchronized dose delivery system for ion therapy which carries the benefits of tumor tracking without the difficulties of implementation (169). In their study, Lis et al. created a 3D treatment plan on each of the 4D breathing phases. With anatomic motion monitoring, dynamic switching between plan libraries for tracking dose delivery was realized through an adaptive layer of software and hardware interfaces. Preliminary tests revealed acceptable dosimetric performance and safety characteristics. The system has the potential to deliver conformal, motion-compensated doses to the moving target.

Various novel beam setups have been proposed to aid in charged particle motion management. The use of a dual carbon-helium ion beam was proposed for carbon ion treatment with helium-guided imaging for range verification (170, 171). The rationale is that for a given energy, helium ions exhibit a range about three times that of carbon ions. This, in conjunction with the fact that both ions have approximately the same charge/mass ratio, means that both ions could be accelerated together in a synchrotron and delivered simultaneously, with the carbon depositing dose in the patient and the helium passing through the patient to a detector to provide online range estimation. Simulation studies using mixed beams have demonstrated the ability to detect range changes as small as 1 mm and detect changes in simulated rotations and bowel gas movement in anthropomorphic phantoms (172). Another novel beam delivery method is the use of radioactive ion treatment beams such as ^11^C and ^15^O, which could be utilized for improved PET imaging to achieve more accurate online range verification (173). Finally, patient orientation with respect to the ion beam can also be considered for reducing motion. Patient setup using an upright treatment posture has also been shown to reduce thoracic motion while increasing lung volume, and has been suggested as a potential motion mitigation strategy for lung cancer patients who cannot tolerate DIBH (174). A limitation of upright radiotherapy treatment posture is that for patient simulation it necessitates using a vertical CT to ensure accurate localization of anatomy between simulation and treatment; currently upright treatment of charged ion beams is limited to only a few centers (175).

Just as the evolution of proton therapy from passive scattering to scanned beam delivery presented new challenges in handling patient motion, future advancements in charged particle treatment delivery will necessitate new innovations to ensure patient safety in the face of motion. One promising emerging radiation technology is FLASH radiation therapy. Defined as radiation therapy delivered in ultra-high dose rates (>40Gy/s), FLASH RT has been shown for *in vivo* animal models to achieve less toxicity for normal tissues while providing the same degree of tumor control (176–179). Multiple platforms (180) including proton clinical machines (181, 182) were able to generate FLASH beams, but biological and technological challenges remain to realize clinical FLASH delivery. Further work on understanding the FLASH mechanisms, as well as optimal treatment dose, dose rates, and fractionation scheme needs to be established (183–185). Shortened treatment delivery times resulting from FLASH dose rates would potentially alleviate concerns about intra-fraction motion, but this also means that any unaccounted-for setup uncertainties and motion will likely lead to the dose being delivered to a completely unexpected location with no opportunity to be mitigated. Thus, the FLASH treatment paradigm necessitates a revisit on current clinical motion management practice and the exploration on the limits to ensure safe and accurate dose delivery when considering the “snap” of motion during ultra-fast dose deliveries.

Arc therapy is an emerging technology in proton therapy in which the gantry is rotated continuously through a pre-selected arc during treatment delivery. ARC therapy for photons (VMAT) is a standard clinical tool with several different commercial systems available (186). In 2021, the commercial proton therapy vendor IBA announced a global DynamicARC consortium, signaling a shared commitment between industry and select clinical centers towards translating proton arc therapy into a commercially available system in the near future (187). While proton ARC therapy has yet to be adopted into standard clinical practice as of yet, its application has been investigated and shown promise in reducing treatment delivery times, improving dose conformality, and reducing dose to OARs in multiple treatment sites (188–190). Planning study was also performed for carbon-ion beams to show normal tissue sparing and mitigation on the hypoxia-related tumor radioresistance (191). Robustness against range uncertainties using proton ARC therapy was demonstrated (192, 193). However, a potential drawback of proton ARC therapy is that it results in increased low dose/low LET dose to normal tissues, which could increase the risk of secondary cancers (194). Different treatment delivery scheme and dose distribution in ARC therapy from traditional PBS delivery will need further studies to assess potential interplay between patient motion and the rotating treatment beam.

### Artificial Intelligence

Artificial intelligence (AI) refers to technologies or machines which can perform tasks/calculations with human-like skill and cognition. Machine learning (ML) is a subcategory of AI relating to computer algorithms which can “learn” to perform a task without being explicitly programmed to do so. Machine learning, and in particular deep learning (DL), has found tremendous success in the past decade in many diverse fields, ranging from finance to automotive technologies, and to medicine (195). DL (also called deep machine learning) is a specific class of ML algorithms which is capable of learning higher-order abstract representations or “features” from raw input data, as opposed to classical ML algorithms which learn patterns from user defined (“handcrafted”) features (196). There is no known theoretical basis for the required structure of an algorithm to achieve DL; in practice however, artificial neural networks, which by design perform a nonlinear mapping from input data to the desired output space, have achieved the best success to date. In radiation oncology, machine learning and deep learning have seen exponential growth in applications and are poised to revolutionize the field (197).

Deep learning applications have been investigated for inter-fractional motion management in charged particle therapy. Van der Heyden et al. presented a single detector, multi-energy proton radiography system which relied on artificial intelligence to filter out proton scatter (198). In addition, multiple studies have been reported which utilize deep learning as a tool to facilitate accurate proton dose calculation from daily CBCT images (121, 199–201). Elmahdy et al. also reported on a convolutional neural network (CNN)-based model for robust, automatic contour propagation in prostate cancer for online adaptive proton therapy (202).

Relating to intra-fractional motion management, many studies have been published which apply deep machine learning towards challenges of real-time target tracking for both charged particles as well as photons. For example, Hirai et al. introduced a CNN-based model for marker-less real-time tumor tracking which was tested retrospectively on lung and liver cancer patients who received fluoroscopic imaging during carbon ion therapy (203). Mylonas et al. reported on the use of another CNN-based architecture for real-time tracking of arbitrarily shaped fiducial markers in fluoroscopy images (204). The motivation behind this study was to allow for fiducial marker segmentation without the need for prior maker-characterization, sparing the patient of additional imaging dose. Zhao et al. proposed a deep learning-based method for pancreatic tumor localization without the use of fiducial markers (205). Kim et al. introduced a CNN-based deep learning model for predict future motion of proton therapy patients based on facial expressions (206). In another promising study, Lin et al. describe a “super-learner” model which combined traditional neural networks with decision tree-based ML algorithms to predict the range of patient thoracic motion during proton therapy based on initial diagnostic CT and EMR data (207).

A substantial roadblock to implementing deep learning models in the clinical workflow stems from issues relating to model interpretability. Specifically, the inherent complexity of deep learning algorithms makes it difficult to understand how the models think (hence why DL models are often referred to as “black boxes”) (208). Though capable of high accuracy predictions, DL models are still sensitive to unintended bias in the training data. The ability to understand and interpret the results of clinical models is critical for ensuring patient safety and quality of care in the event that such models fail (209). To this end, the task of developing interpretable machine learning models without sacrificing model performance is an active research topic (210–212). One approach in handling the ethical question of what role a non-human entity should be trusted to play in clinical decision making is to require algorithms to follow a “human in the loop” framework. In a human-in-the-loop framework, clinicians remain fully integrated in the decision making process by allowing for human-computer interactions by which clinicians provide feedback/information to aid in model development, and the resulting models in turn provide accountable, interpretable decision support to the clinician (208).

## Summary

In this review, we provided an overview of the current clinical treatment methods for motion management in charged particle therapy as well as future emerging technologies and methodologies. As the use of charged particle therapy becomes more widespread and new treatment delivery modalities arise, further work will be necessary to ensure robust and accurate dose delivery. For the case of inter-fractional motion, further progress is also necessary to develop adaptive planning protocols which are triggered for clinically significant anatomical changes and can be implemented within the time and resource constraints of a clinical setting. Another important task in this field is to continue the development of imaging and tracking systems which allow for accurate localization of the tumor and internal anatomy delivery at the treatment couch prior to delivery and in real time. One limitation of most studies on motion mitigation methods is that dosimetry is used the primary evaluation metric. Future studies which can connect motion mitigation methods to clinical outcomes would aid in accelerating standardization of care.

## Author Contributions

JP and WZ drafted the manuscript. AK, LD, and AR contributed with discussions, coordination, and critical revision of the manuscript. All authors contributed to the article and approved the submitted version.

## Conflict of Interest

The authors declare that the research was conducted in the absence of any commercial or financial relationships that could be construed as a potential conflict of interest.

## Publisher’s Note

All claims expressed in this article are solely those of the authors and do not necessarily represent those of their affiliated organizations, or those of the publisher, the editors and the reviewers. Any product that may be evaluated in this article, or claim that may be made by its manufacturer, is not guaranteed or endorsed by the publisher.

## References

[1] Newhauser WD Zhang R. The Physics of Proton Therapy. Phys Med Biol (2015) 60(8):R155–209. doi: 10.1088/0031-9155/60/8/R155 PMC4407514 25803097

[2] Chang JY Zhang X Wang X Kang Y Riley B Bilton S. Significant Reduction of Normal Tissue Dose by Proton Radiotherapy Compared With Three-Dimensional Conformal or Intensity-Modulated Radiation Therapy in Stage I or Stage III Non-Small-Cell Lung Cancer. Int J Radiat Oncol Biol Phys (2006) 65(4):1087–96. doi: 10.1016/j.ijrobp.2006.01.052 16682145

[3] van de Water TA Lomax AJ Bijl HP de Jong ME Schilstra C Hug EB. Potential Benefits of Scanned Intensity-Modulated Proton Therapy Versus Advanced Photon Therapy With Regard to Sparing of the Salivary Glands in Oropharyngeal Cancer. Int J Radiat Oncol Biol Phys (2011) 79(4):1216–24. doi: 10.1016/j.ijrobp.2010.05.012 20732761

[4] Loeffler JS Durante M. Charged Particle Therapy—Optimization, Challenges and Future Directions. Nat Rev Clin Oncol (2013) 10(7):411–24. doi: 10.1038/nrclinonc.2013.79 23689752

[5] De Ruysscher D Chang JY. Clinical Controversies: Proton Therapy for Thoracic Tumors. Semin Radiat Oncol (2013) 23(2):115–9. doi: 10.1016/j.semradonc.2012.11.010 23473688

[6] Durante M Orecchia R Loeffler JS. Charged-Particle Therapy in Cancer: Clinical Uses and Future Perspectives. Nat Rev Clin Oncol (2017) 14(8):483–95. doi: 10.1038/nrclinonc.2017.30 28290489

[7] Lomax AJ. Intensity Modulated Proton Therapy and its Sensitivity to Treatment Uncertainties 2: The Potential Effects of Inter-Fraction and Inter-Field Motions. Phys Med Biol (2008) 53(4):1043–56. doi: 10.1088/0031-9155/53/4/015 18263957

[8] Particle Therapy Facilities in Clinical Operation (Last Update: (October 2021) [Internet] (2021). Available at: https://www.ptcog.ch/index.php/facilities-in-operation.

[9] Chang JY Zhang X Knopf A Li H Mori S Dong L. Consensus Guidelines for Implementing Pencil-Beam Scanning Proton Therapy for Thoracic Malignancies on Behalf of the PTCOG Thoracic and Lymphoma Subcommittee. Int J Radiat Oncol Biol Phys (2017) 99(1):41–50. doi: 10.1016/j.ijrobp.2017.05.014 28816159

[10] Madani I Lomax AJ Albertini F Trnková P Weber DC. Dose-Painting Intensity-Modulated Proton Therapy for Intermediate- and High-Risk Meningioma. Radiat Oncol (2015) 10:72. doi: 10.1186/s13014-015-0384-x 25890217 PMC4404662

[11] Welsh J Gomez D Palmer MB Riley BA Mayankkumar AV Komaki R. Intensity-Modulated Proton Therapy Further Reduces Normal Tissue Exposure During Definitive Therapy for Locally Advanced Distal Esophageal Tumors: A Dosimetric Study. Int J Radiat Oncol Biol Phys (2011) 81(5):1336–42. doi: 10.1016/j.ijrobp.2010.07.2001 PMC4086056 21470796

[12] Shiraishi Y Xu C Yang J Komaki R Lin SH. Dosimetric Comparison to the Heart and Cardiac Substructure in a Large Cohort of Esophageal Cancer Patients Treated With Proton Beam Therapy or Intensity-Modulated Radiation Therapy. Radiother Oncol (2017) 125(1):48–54. doi: 10.1016/j.radonc.2017.07.034 28917586

[13] Bhangoo RS Mullikin TC Ashman JB Cheng TW Golafshar MA DeWees TA. Intensity Modulated Proton Therapy for Hepatocellular Carcinoma: Initial Clinical Experience. Adv Radiat Oncol (2021) 6(4). doi: 10.1016/j.adro.2021.100675 PMC8361033 34409199

[14] Wang X Krishnan S Zhang X Dong L Briere T Crane CH. Proton Radiotherapy for Liver Tumors: Dosimetric Advantages Over Photon Plans. Med Dosim (2008) 33(4):259–67. doi: 10.1016/j.meddos.2007.04.008 18973852

[15] Bert C Herfarth K. Management of Organ Motion in Scanned Ion Beam Therapy. Radiat Oncol (2017) 12:170. doi: 10.1186/s13014-017-0911-z 29110693 PMC5674859

[16] Grassberger C Dowdell S Lomax A Sharp G Shackleford J Choi N. Motion Interplay as a Function of Patient Parameters and Spot Size in Spot Scanning Proton Therapy for Lung Cancer. Int J Radiat Oncol Biol Phys (2013) 86(2):380–6. doi: 10.1016/j.ijrobp.2013.01.024 PMC3646997 23462423

[17] Møller DS Poulsen PR Hagner A Dufour M Nordsmark M Nyeng TB. Strategies for Motion Robust Proton Therapy With Pencil Beam Scanning for Esophageal Cancer. Int J Radiat Oncol Biol Phys (2021) 111(2):539–48. doi: 10.1016/j.ijrobp.2021.04.040 33974885

[18] Kraus KM Heath E Oelfke U. Dosimetric Consequences of Tumour Motion Due to Respiration for a Scanned Proton Beam. Phys Med Biol (2011) 56(20):6563–81. doi: 10.1088/0031-9155/56/20/003 21937770

[19] Zhang Y Huth I Weber DC Lomax AJ. Dosimetric Uncertainties as a Result of Temporal Resolution in 4D Dose Calculations for PBS Proton Therapy. Phys Med Biol (2019) 64(12):125005. doi: 10.1088/1361-6560/ab1d6f 31035271

[20] Schippers JM. Beam Delivery Systems for Particle Radiation Therapy: Current Status and Recent Developments. Rev Accelerator Sci Technol (2009) 02(01):179–200. doi: 10.1142/9789814299350_0009

[21] Zhang Y Huth I Weber DC Lomax AJ. A Statistical Comparison of Motion Mitigation Performances and Robustness of Various Pencil Beam Scanned Proton Systems for Liver Tumour Treatments. Radiother Oncol (2018) 128(1):182–8. doi: 10.1016/j.radonc.2018.01.019 29459153

[22] Group PTC-O. Particle Therapy Facilities Under Construction. (2021) Available at: https://www.ptcog.ch/index.php/facilities-under-construction.

[23] Tsujii H Kamada T Shirai T Noda K Tsuji H Karasawa K. Carbon-Ion Radiotherapy Principles, Practices, and Treatment Planning. 1st ed Vol. 2014. Springer Japan: Imprint: Springer (2014). p. 2014.

[24] Malouff TD Mahajan A Krishnan S Beltran C Seneviratne DS Trifiletti DM. Carbon Ion Therapy: A Modern Review of an Emerging Technology. Front Oncol (2020) 10(82). doi: 10.3389/fonc.2020.00082 PMC7010911 32117737

[25] Krämer M Scholz M. Treatment Planning for Heavy-Ion Radiotherapy: Calculation and Optimization of Biologically Effective Dose. Phys Med Biol (2000) 45(11):3319–30. doi: 10.1088/0031-9155/45/11/314 11098906

[26] Paganetti H Botas P Sharp GC Winey B. Adaptive Proton Therapy. Phys Med Biol (2021) 66(22). doi: 10.1088/1361-6560/ac344f PMC8628198 34710858

[27] Wang X Yu M Wang J Zhong R Shen Y Zhao Y. An Assessment of Interfractional Bladder, Rectum and Vagina Motion in Postoperative Cervical Cancer Based on Daily Cone-Beam Computed Tomography. Mol Clin Oncol (2016) 4(2):271–7. doi: 10.3892/mco.2015.704 PMC4734198 26893874

[28] Chen L Paskalev K Xu X Zhu J Wang L Price RA. Rectal Dose Variation During the Course of Image-Guided Radiation Therapy of Prostate Cancer. Radiother Oncol (2010) 95(2):198–202. doi: 10.1016/j.radonc.2010.02.023 20303193

[29] Bolsi A Peroni M Amelio D Dasu A Stock M Toma-Dasu I. Practice Patterns of Image Guided Particle Therapy in Europe: A 2016 Survey of the European Particle Therapy Network (EPTN). Radiother Oncol (2018) 128(1):4–8. doi: 10.1016/j.radonc.2018.03.017 29605478

[30] Dueck J Knopf AC Lomax A Albertini F Persson GF Josipovic M. Robustness of the Voluntary Breath-Hold Approach for the Treatment of Peripheral Lung Tumors Using Hypofractionated Pencil Beam Scanning Proton Therapy. Int J Radiat Oncol Biol Phys (2016) 95(1):534–41. doi: 10.1016/j.ijrobp.2015.11.015 26797540

[31] Wong JW Sharpe MB Jaffray DA Kini VR Robertson JM Stromberg JS. The Use of Active Breathing Control (ABC) to Reduce Margin for Breathing Motion. Int J Radiat Oncol Biol Phys (1999) 44(4):911–9. doi: 10.1016/S0360-3016(99)00056-5 10386650

[32] Baues C Marnitz S Engert A Baus W Jablonska K Fogliata A. Proton Versus Photon Deep Inspiration Breath Hold Technique in Patients With Hodgkin Lymphoma and Mediastinal Radiation: A PLANNING COMPARISON OF DEEP INSPIRATION BREATH HOLD INTENSITY MODULATION RADIOTHERAPY AND INTENSITY MODULATED PROTON THERAPY. Radiat Oncol (2018) 13(1):122. doi: 10.1186/s13014-018-1066-2 29970105 PMC6029162

[33] Mondal D Jhawar SR Millevoi R Haffty BG Parikh RR. Proton Versus Photon Breath-Hold Radiation for Left-Sided Breast Cancer After Breast-Conserving Surgery: A Dosimetric Comparison. Int J Part Ther (2020) 7(3):24–33. doi: 10.14338/IJPT-20-00026.1 33604413 PMC7886268

[34] Lee HL Lim LH Master Z Wong SMM. The Role of Breath Hold Intensity Modulated Proton Therapy for a Case of Left-Sided Breast Cancer With IMN Involvement. How Protons Compare With Other Conformal Techniques? Tech Innov Patient Support Radiat Oncol (2020) 15:1–5. doi: 10.1016/j.tipsro.2020.03.001 32490219 PMC7256639

[35] Emert F Missimer J Eichenberger PA Walser M Gmür C Lomax AJ. Enhanced Deep-Inspiration Breath Hold Superior to High-Frequency Percussive Ventilation for Respiratory Motion Mitigation: A Physiology-Driven, MRI-Guided Assessment Toward Optimized Lung Cancer Treatment With Proton Therapy. Front Oncol (2021) 11:621350. doi: 10.3389/fonc.2021.621350 PMC8116693 33996545

[36] Mori S Lu HM Wolfgang JA Choi NC Chen GT. Effects of Interfractional Anatomical Changes on Water-Equivalent Pathlength in Charged-Particle Radiotherapy of Lung Cancer. J Radiat Res (2009) 50(6):513–9. doi: 10.1269/jrr.09032 19959880

[37] Lin L Souris K Kang M Glick A Lin H Huang S. Evaluation of Motion Mitigation Using Abdominal Compression in the Clinical Implementation of Pencil Beam Scanning Proton Therapy of Liver Tumors. Med Phys (2017) 44(2):703–12. doi: 10.1002/mp.12040 28133755

[38] Taunk NK Burgdorf B Dong L Ben-Josef E. Simultaneous Multiple Liver Metastasis Treated With Pencil Beam Proton Stereotactic Body Radiotherapy (SBRT). Int J Part Ther (2021) 8(2):89–94. doi: 10.14338/IJPT-20-00085.1 34722815 PMC8489493

[39] Meschini G Seregni M Pella A Ciocca M Fossati P Valvo F. Evaluation of Residual Abdominal Tumour Motion in Carbon Ion Gated Treatments Through Respiratory Motion Modelling. Physica Med (2017) 34:28–37. doi: 10.1016/j.ejmp.2017.01.009 28109567

[40] Eminowicz G Motlib J Khan S Perna C McCormack M. Pelvic Organ Motion During Radiotherapy for Cervical Cancer: Understanding Patterns and Recommended Patient Preparation. Clin Oncol (R Coll Radiol) (2016) 28(9):e85–91. doi: 10.1016/j.clon.2016.04.044 27178706

[41] Wroe AJ Bush DA Schulte RW Slater JD. Clinical Immobilization Techniques for Proton Therapy. Technol Cancer Res Treat (2015) 14(1):71–9. doi: 10.7785/tcrt.2012.500398 24354755

[42] Teh BS McGary JE Dong L Mai W-Y Carpenter LS Lu HH. The Use of Rectal Balloon During the Delivery of Intensity Modulated Radiotherapy (IMRT) for Prostate Cancer: More Than Just a Prostate Gland Immobilization Device? Cancer J (2002) 8(6). doi: 10.1097/00130404-200211000-00039 12500857

[43] Vargas C Mahajan C Fryer A Indelicato D Henderson RH McKenzie C. Rectal Dose–Volume Differences Using Proton Radiotherapy and a Rectal Balloon or Water Alone for the Treatment of Prostate Cancer. Int J Radiat Oncol Biol Phys (2007) 69(4):1110–6. doi: 10.1016/j.ijrobp.2007.04.075 17967305

[44] Dinh TT Lee HJ Jr. Macomber MW Apisarnthanarax S Zeng J Laramore GE. Rectal Hydrogel Spacer Improves Late Gastrointestinal Toxicity Compared to Rectal Balloon Immobilization After Proton Beam Radiation Therapy for Localized Prostate Cancer: A Retrospective Observational Study. Int J Radiat Oncol Biol Phys (2020) 108(3):635–43. doi: 10.1016/j.ijrobp.2020.01.026 32035187

[45] Balter JM Ten Haken RK Lawrence TS Lam KL Robertson JM. Uncertainties in CT-Based Radiation Therapy Treatment Planning Associated With Patient Breathing. Int J Radiat Oncol Biol Phys (1996) 36(1):167–74. doi: 10.1016/S0360-3016(96)00275-1 8823272

[46] Wolthaus JWH Schneider C Sonke J-J van Herk M Belderbos JSA Rossi MMG. Mid-Ventilation CT Scan Construction From Four-Dimensional Respiration-Correlated CT Scans for Radiotherapy Planning of Lung Cancer Patients. Int J Radiat Oncol Biol Phys (2006) 65(5):1560–71. doi: 10.1016/j.ijrobp.2006.04.031 16863933

[47] Sindoni A Minutoli F Pontoriero A Iatì G Baldari S Pergolizzi S. Usefulness of Four Dimensional (4D) PET/CT Imaging in the Evaluation of Thoracic Lesions and in Radiotherapy Planning: Review of the Literature. Lung Cancer (2016) 96:78–86. doi: 10.1016/j.lungcan.2016.03.019 27133755

[48] Krieger M Giger A Salomir R Bieri O Celicanin Z Cattin PC. Impact of Internal Target Volume Definition for Pencil Beam Scanned Proton Treatment Planning in the Presence of Respiratory Motion Variability for Lung Cancer: A Proof of Concept. Radiother Oncol (2020) 145:154–61. doi: 10.1016/j.radonc.2019.12.001 32007759

[49] Li G Liu Y Nie X. Respiratory-Correlated (RC) vs. Time-Resolved (TR) Four-Dimensional Magnetic Resonance Imaging (4DMRI) for Radiotherapy of Thoracic and Abdominal Cancer. Front Oncol (2019) 9:1024. doi: 10.3389/fonc.2019.01024 PMC6798178 31681573

[50] Rabe M Thieke C Düsberg M Neppl S Gerum S Reiner M. Real-Time 4DMRI-Based Internal Target Volume Definition for Moving Lung Tumors. Med Phys (2020) 47(4):1431–42. doi: 10.1002/mp.14023 31955430

[51] Dolde K Zhang Y Chaudhri N Dávid C Kachelrieß M Lomax AJ. 4DMRI-Based Investigation on the Interplay Effect for Pencil Beam Scanning Proton Therapy of Pancreatic Cancer Patients. Radiat Oncol (2019) 14(1):30. doi: 10.1186/s13014-019-1231-2 30732657 PMC6367829

[52] Veiga C Janssens G Teng C-L Baudier T Hotoiu L McClelland JR. First Clinical Investigation of Cone Beam Computed Tomography and Deformable Registration for Adaptive Proton Therapy for Lung Cancer. Int J Radiat Oncol Biol Phys (2016) 95(1):549–59. doi: 10.1016/j.ijrobp.2016.01.055 27084664

[53] Landry G Nijhuis R Dedes G Handrack J Thieke C Janssens G. Investigating CT to CBCT Image Registration for Head and Neck Proton Therapy as a Tool for Daily Dose Recalculation. Med Phys (2015) 42(3):1354–66. doi: 10.1118/1.4908223 25735290

[54] Park YK Sharp GC Phillips J Winey BA. Proton Dose Calculation on Scatter-Corrected CBCT Image: Feasibility Study for Adaptive Proton Therapy. Med Phys (2015) 42(8):4449–59. doi: 10.1118/1.4923179 PMC4517932 26233175

[55] Kurz C Kamp F Park YK Zöllner C Rit S Hansen D. Investigating Deformable Image Registration and Scatter Correction for CBCT-Based Dose Calculation in Adaptive IMPT. Med Phys (2016) 43(10):5635. doi: 10.1118/1.4962933 27782706

[56] Veiga C Janssens G Baudier T Hotoiu L Brousmiche S McClelland J. A Comprehensive Evaluation of the Accuracy of CBCT and Deformable Registration Based Dose Calculation in Lung Proton Therapy. Biomed Phys Eng Express (2017) 3(1):015003. doi: 10.1088/2057-1976/3/1/015003

[57] Nesteruk KP Bobić M Lalonde A Winey BA Lomax AJ Paganetti H. CT-On-Rails Versus In-Room CBCT for Online Daily Adaptive Proton Therapy of Head-And-Neck Cancers. Cancers (Basel) (2021) 13(23):5991. doi: 10.3390/cancers13235991 34885100 PMC8656713

[58] Landry G Hua C-h. Current State and Future Applications of Radiological Image Guidance for Particle Therapy. Med Phys (2018) 45(11):e1086–e95. doi: 10.1002/mp.12744 30421805

[59] Sawant A Smith RL Venkat RB Santanam L Cho B Poulsen P. Geometric Accuracy and Latency of an Integrated 4d IMRT Delivery System Using Real-Time Internal Position Monitoring and Dynamic MLC Tracking. Int J Radiat Oncol Biol Phys (2008) 72(1):S27–S8. doi: 10.1016/j.ijrobp.2008.06.828

[60] Ehrbar S Perrin R Peroni M Bernatowicz K Parkel T Pytko I. Respiratory Motion-Management in Stereotactic Body Radiation Therapy for Lung Cancer - A Dosimetric Comparison in an Anthropomorphic Lung Phantom (LuCa). Radiother Oncol (2016) 121(2):328–34. doi: 10.1016/j.radonc.2016.10.011 27817945

[61] Fattori G Safai S Carmona PF Peroni M Perrin R Weber DC. Monitoring of Breathing Motion in Image-Guided PBS Proton Therapy: Comparative Analysis of Optical and Electromagnetic Technologies. Radiat Oncol (2017) 12(1):63–. doi: 10.1186/s13014-017-0797-9 PMC5374699 28359341

[62] Mao W Wiersma RD Xing L. Fast Internal Marker Tracking Algorithm for Onboard MV and kV Imaging Systems. Med Phys (2008) 35(5):1942–9. doi: 10.1118/1.2905225 PMC2809731 18561670

[63] Tang S Deville C Tochner Z Wang KK McDonough J Vapiwala N. Impact of Intrafraction and Residual Interfraction Effect on Prostate Proton Pencil Beam Scanning. Int J Radiat Oncol Biol Phys (2014) 90(5):1186–94. doi: 10.1016/j.ijrobp.2014.08.015 25442043

[64] Reidel C-A Schuy C Horst F Ecker S Finck C Durante M. Fluence Perturbation From Fiducial Markers Due to Edge-Scattering Measured With Pixel Sensors for 12C Ion Beams. Phys Med Biol (2020) 65(8):085005. doi: 10.1088/1361-6560/ab762f 32053811

[65] Shirato H Shimizu S Kunieda T Kitamura K van Herk M Kagei K. Physical Aspects of a Real-Time Tumor-Tracking System for Gated Radiotherapy. Int J Radiat Oncol Biol Phys (2000) 48(4):1187–95. doi: 10.1016/S0360-3016(00)00748-3 11072178

[66] Shimizu S Miyamoto N Matsuura T Fujii Y Umezawa M Umegaki K. A Proton Beam Therapy System Dedicated to Spot-Scanning Increases Accuracy With Moving Tumors by Real-Time Imaging and Gating and Reduces Equipment Size. PloS One (2014) 9(4):e94971. doi: 10.1371/journal.pone.0094971 24747601 PMC3991640

[67] Yoshimura T Shimizu S Hashimoto T Nishioka K Katoh N Inoue T. Analysis of Treatment Process Time for Real-Time-Image Gated-Spot-Scanning Proton-Beam Therapy (RGPT) System. J Appl Clin Med Phys (2020) 21(2):38–49. doi: 10.1002/acm2.12804 PMC7020995 31886616

[68] Shirato H Harada T Harabayashi T Hida K Endo H Kitamura K. Feasibility of Insertion/Implantation of 2.0-Mm-Diameter Gold Internal Fiducial Markers for Precise Setup and Real-Time Tumor Tracking in Radiotherapy. Int J Radiat Oncol Biol Phys (2003) 56(1):240–7. doi: 10.1016/s0360-3016(03)00076-2 12694845

[69] Freislederer P Kügele M Öllers M Swinnen A Sauer TO Bert C. Recent Advances in Surface Guided Radiation Therapy. Radiat Oncol (2020) 15(1):187. doi: 10.1186/s13014-020-01629-w 32736570 PMC7393906

[70] MacFarlane MJ Jiang K Mundis M Nichols E Gopal A Chen S. Comparison of the Dosimetric Accuracy of Proton Breast Treatment Plans Delivered With SGRT and CBCT Setups. J Appl Clin Med Phys (2021) 22(9):153–8. doi: 10.1002/acm2.13357 PMC8425866 34288378

[71] Batin E Depauw N Jimenez RB MacDonald S Lu HM. Reducing X-Ray Imaging for Proton Postmastectomy Chest Wall Patients. Pract Radiat Oncol (2018) 8(5):e266–e74. doi: 10.1016/j.prro.2018.03.002 29907510

[72] Depauw N Batin E Daartz J Rosenfeld A Adams J Kooy H. A Novel Approach to Postmastectomy Radiation Therapy Using Scanned Proton Beams. Int J Radiat Oncol Biol Phys (2015) 91(2):427–34. doi: 10.1016/j.ijrobp.2014.10.039 25636765

[73] Walter F Freislederer P Belka C Heinz C Söhn M Roeder F. Evaluation of Daily Patient Positioning for Radiotherapy With a Commercial 3D Surface-Imaging System (Catalyst™). Radiat Oncol (2016) 11(1):154. doi: 10.1186/s13014-016-0728-1 27881158 PMC5122202

[74] Carl G Reitz D Schönecker S Pazos M Freislederer P Reiner M. Optical Surface Scanning for Patient Positioning in Radiation Therapy: A Prospective Analysis of 1902 Fractions. Technol Cancer Res Treat (2018) 17:1533033818806002. doi: 10.1177/1533033818806002 30453842 PMC6243634

[75] Stanley DN McConnell KA Kirby N Gutiérrez AN Papanikolaou N Rasmussen K. Comparison of Initial Patient Setup Accuracy Between Surface Imaging and Three Point Localization: A Retrospective Analysis. J Appl Clin Med Phys (2017) 18(6):58–61. doi: 10.1002/acm2.12183 28901684 PMC5689923

[76] Czerska K Emert F Kopec R Langen K McClelland JR Meijers A. Clinical Practice vs. State-of-the-Art Research and Future Visions: Report on the 4D Treatment Planning Workshop for Particle Therapy - Edition 2018 and 2019. Phys Med (2021) 82:54–63. doi: 10.1016/j.ejmp.2020.12.013 33588228

[77] Knopf A Bert C Heath E Nill S Kraus K Richter D. Special Report: Workshop on 4D-Treatment Planning in Actively Scanned Particle Therapy—Recommendations, Technical Challenges, and Future Research Directions. Med Phys (2010) 37(9):4608–14. doi: 10.1118/1.3475944 20964178

[78] Kang Y Zhang X Chang JY Wang H Wei X Liao Z. 4d Proton Treatment Planning Strategy for Mobile Lung Tumors. Int J Radiat Oncol Biol Phys (2007) 67(3):906–14. doi: 10.1016/j.ijrobp.2006.10.045 17293240

[79] Visser S Neh H Oraboni Ribeiro C Korevaar EW Meijers A Poppe B. Assessment of a Diaphragm Override Strategy for Robustly Optimized Proton Therapy Planning for Esophageal Cancer Patients. Med Phys (2021) n/a(n/a). doi: 10.1002/mp.15114 PMC9291176 34289123

[80] van Herk M Remeijer P Rasch C Lebesque JV. The Probability of Correct Target Dosage: Dose-Population Histograms for Deriving Treatment Margins in Radiotherapy. Int J Radiat Oncol Biol Phys (2000) 47(4):1121–35. doi: 10.1016/S0360-3016(00)00518-6 10863086

[81] Phillips MH Pedroni E Blattmann H Boehringer T Coray A Scheib S. Effects of Respiratory Motion on Dose Uniformity With a Charged Particle Scanning Method. Phys Med Biol (1992) 37(1):223–34. doi: 10.1088/0031-9155/37/1/016 1311106

[82] Seco J Robertson D Trofimov A Paganetti H. Breathing Interplay Effects During Proton Beam Scanning: Simulation and Statistical Analysis. Phys Med Biol (2009) 54(14):N283–94. doi: 10.1088/0031-9155/54/14/N01 19550002

[83] Zhang Y Huth I Wegner M Weber DC Lomax AJ. An Evaluation of Rescanning Technique for Liver Tumour Treatments Using a Commercial PBS Proton Therapy System. Radiother Oncol (2016) 121(2):281–7. doi: 10.1016/j.radonc.2016.09.011 27726957

[84] Kardar L Li Y Li X Li H Cao W Chang JY. Evaluation and Mitigation of the Interplay Effects of Intensity Modulated Proton Therapy for Lung Cancer in a Clinical Setting. Pract Radiat Oncol (2014) 4(6):e259–e68. doi: 10.1016/j.prro.2014.06.010 PMC4399385 25407877

[85] Poulsen PR Eley J Langner U Simone CB Langen K. Efficient Interplay Effect Mitigation for Proton Pencil Beam Scanning by Spot-Adapted Layered Repainting Evenly Spread Out Over the Full Breathing Cycle. Int J Radiat Oncol Biol Phys (2018) 100(1):226–34. doi: 10.1016/j.ijrobp.2017.09.043 29254775

[86] Gelover E Deisher AJ Herman MG Johnson JE Kruse JJ Tryggestad EJ. Clinical Implementation of Respiratory-Gated Spot-Scanning Proton Therapy: An Efficiency Analysis of Active Motion Management. J Appl Clin Med Phys (2019) 20(5):99–108. doi: 10.1002/acm2.12584 PMC6523004 30972922

[87] Nakajima K Iwata H Ogino H Hattori Y Hashimoto S Toshito T. Clinical Outcomes of Image-Guided Proton Therapy for Histologically Confirmed Stage I non-Small Cell Lung Cancer. Radiat Oncol (2018) 13(1):199. doi: 10.1186/s13014-018-1144-5 30305125 PMC6180633

[88] Ebner DK Tsuji H Yasuda S Yamamoto N Mori S Kamada T. Respiration-Gated Fast-Rescanning Carbon-Ion Radiotherapy. Jpn J Clin Oncol (2017) 47(1):80–3. doi: 10.1093/jjco/hyw144 27677663

[89] Mori S Karube M Shirai T Tajiri M Takekoshi T Miki K. Carbon-Ion Pencil Beam Scanning Treatment With Gated Markerless Tumor Tracking: An Analysis of Positional Accuracy. Int J Radiat Oncol Biol Phys (2016) 95(1):258–66. doi: 10.1016/j.ijrobp.2016.01.014 26960747

[90] Zhang M Zou W Teo B-KK. Image Guidance in Proton Therapy for Lung Cancer. Trans Lung Cancer Res (2018) 7(2). doi: 10.21037/tlcr.2018.03.26 PMC5960660 29876315

[91] Matsuura T Miyamoto N Shimizu S Fujii Y Umezawa M Takao S. Integration of a Real-Time Tumor Monitoring System Into Gated Proton Spot-Scanning Beam Therapy: An Initial Phantom Study Using Patient Tumor Trajectory Data. Med Phys (2013) 40(7):071729. doi: 10.1118/1.4810966 23822433

[92] Bert C Gemmel A Saito N Rietzel E. Gated Irradiation With Scanned Particle Beams. Int J Radiat OncologyBiologyPhysics (2009) 73(4):1270–5. doi: 10.1016/j.ijrobp.2008.11.014 19251099

[93] Furukawa T Inaniwa T Sato S Tomitani T Minohara S Noda K. Design Study of a Raster Scanning System for Moving Target Irradiation in Heavy-Ion Radiotherapy. Med Phys (2007) 34(3):1085–97. doi: 10.1118/1.2558213 17441254

[94] Zhang Y Knopf A-C Weber DC Lomax AJ. Improving 4D Plan Quality for PBS-Based Liver Tumour Treatments by Combining Online Image Guided Beam Gating With Rescanning. Phys Med Biol (2015) 60(20):8141–59. doi: 10.1088/0031-9155/60/20/8141 26439493

[95] Schätti A Zakova M Meer D Lomax AJ. The Effectiveness of Combined Gating and Re-Scanning for Treating Mobile Targets With Proton Spot Scanning. An Experimental and Simulation-Based Investigation. Phys Med Biol (2014) 59(14):3813–28. doi: 10.1088/0031-9155/59/14/3813 24955723

[96] Unkelbach J Chan TCY Bortfeld T. Accounting for Range Uncertainties in the Optimization of Intensity Modulated Proton Therapy. Phys Med Biol (2007) 52(10):2755–73. doi: 10.1088/0031-9155/52/10/009 17473350

[97] Unkelbach J Bortfeld T Martin BC Soukup M. Reducing the Sensitivity of IMPT Treatment Plans to Setup Errors and Range Uncertainties *via* Probabilistic Treatment Planning. Med Phys (2009) 36(1):149–63. doi: 10.1118/1.3021139 PMC2673668 19235384

[98] Fredriksson A Forsgren A Hårdemark B. Minimax Optimization for Handling Range and Setup Uncertainties in Proton Therapy. Med Phys (2011) 38(3):1672–84. doi: 10.1118/1.3556559 21520880

[99] Pflugfelder D Wilkens JJ Oelfke U. Worst Case Optimization: A Method to Account for Uncertainties in the Optimization of Intensity Modulated Proton Therapy. Phys Med Biol (2008) 53(6):1689–700. doi: 10.1088/0031-9155/53/6/013 18367797

[100] Ge S Wang X Liao Z Zhang L Sahoo N Yang J. Potential for Improvements in Robustness and Optimality of Intensity-Modulated Proton Therapy for Lung Cancer With 4-Dimensional Robust Optimization. Cancers (Basel) (2019) 11(1):35. doi: 10.3390/cancers11010035 PMC6356681 30609652

[101] Liu W Schild SE Chang JY Liao Z Chang Y-H Wen Z. Exploratory Study of 4D Versus 3D Robust Optimization in Intensity Modulated Proton Therapy for Lung Cancer. Int J Radiat Oncol Biol Phys (2016) 95(1):523–33. doi: 10.1016/j.ijrobp.2015.11.002 PMC4834263 26725727

[102] Mastella E Molinelli S Pella A Vai A Maestri D Vitolo V. 4D Strategies for Lung Tumors Treated With Hypofractionated Scanning Proton Beam Therapy: Dosimetric Impact and Robustness to Interplay Effects. Radiother Oncol (2020) 146:213–20. doi: 10.1016/j.radonc.2020.02.025 32222489

[103] Richter D Saito N Chaudhri N Härtig M Ellerbrock M Jäkel O. Four-Dimensional Patient Dose Reconstruction for Scanned Ion Beam Therapy of Moving Liver Tumors. Int J Radiat Oncol Biol Phys (2014) 89(1):175–81. doi: 10.1016/j.ijrobp.2014.01.043 24725700

[104] Eley JG Newhauser WD Lüchtenborg R Graeff C Bert C. 4D Optimization of Scanned Ion Beam Tracking Therapy for Moving Tumors. Phys Med Biol (2014) 59(13):3431–52. doi: 10.1088/0031-9155/59/13/3431 PMC4139294 24889215

[105] Graeff C Lüchtenborg R Eley JG Durante M Bert C. A 4D-Optimization Concept for Scanned Ion Beam Therapy. Radiother Oncol (2013) 109(3):419–24. doi: 10.1016/j.radonc.2013.09.018 24183865

[106] Wolf M Anderle K Durante M Graeff C. Robust Treatment Planning With 4D Intensity Modulated Carbon Ion Therapy for Multiple Targets in Stage IV non-Small Cell Lung Cancer. Phys Med Biol (2020) 65(21):215012. doi: 10.1088/1361-6560/aba1a3 32610300

[107] Meschini G Kamp F Hofmaier J Reiner M Sharp G Paganetti H. Modeling RBE-Weighted Dose Variations in Irregularly Moving Abdominal Targets Treated With Carbon Ion Beams. Med Phys (2020) 47(7):2768–78. doi: 10.1002/mp.14135 32162332

[108] Bert C Herfarth K. Management of Organ Motion in Scanned Ion Beam Therapy. Radiat Oncol (2017) 12(1):170. doi: 10.1186/s13014-017-0911-z 29110693 PMC5674859

[109] RayStation: Robustness in Treatment Planning: RaySearch Laboratories. Available at: https://www.raysearchlabs.com/robustness-in-treatment-planning/#:~:text=RayStation%20utilizes%20a%20unique%20robust,the%20use%20of%20multiple%20images.

[110] Meijers A Knopf AC Crijns APG Ubbels JF Niezink AGH Langendijk JA. Evaluation of Interplay and Organ Motion Effects by Means of 4D Dose Reconstruction and Accumulation. Radiother Oncol (2020) 150:268–74. doi: 10.1016/j.radonc.2020.07.055 32768509

[111] Meijers A Jakobi A Stützer K Guterres Marmitt G Both S Langendijk JA. Log File-Based Dose Reconstruction and Accumulation for 4D Adaptive Pencil Beam Scanned Proton Therapy in a Clinical Treatment Planning System: Implementation and Proof-of-Concept. Med Phys (2019) 46(3):1140–9. doi: 10.1002/mp.13371 30609061

[112] Ribeiro CO Meijers A Korevaar EW Muijs CT Both S Langendijk JA. Comprehensive 4D Robustness Evaluation for Pencil Beam Scanned Proton Plans. Radiother Oncol (2019) 136:185–9. doi: 10.1016/j.radonc.2019.03.037 31015123

[113] Korevaar EW Habraken SJM Scandurra D Kierkels RGJ Unipan M Eenink MGC. Practical Robustness Evaluation in Radiotherapy – A Photon and Proton-Proof Alternative to PTV-Based Plan Evaluation. Radiother Oncol (2019) 141:267–74. doi: 10.1016/j.radonc.2019.08.005 31492443

[114] Magro G Mein S Kopp B Mastella E Pella A Ciocca M. FRoG Dose Computation Meets Monte Carlo Accuracy for Proton Therapy Dose Calculation in Lung. Physica Med (2021) 86:66–74. doi: 10.1016/j.ejmp.2021.05.021 34058719

[115] Gajewski J Garbacz M Chang C-W Czerska K Durante M Krah N. Commissioning of GPU–Accelerated Monte Carlo Code FRED for Clinical Applications in Proton Therapy. Front Phys (2021) 8:403. doi: 10.3389/fphy.2020.567300

[116] Pepin MD Tryggestad E Wan Chan Tseung HS Johnson JE Herman MG Beltran C. A Monte-Carlo-Based and GPU-Accelerated 4D-Dose Calculator for a Pencil Beam Scanning Proton Therapy System. Med Phys (2018) 45(11):5293–304. doi: 10.1002/mp.13182 30203550

[117] Zhang Y Yin F-F Ren L. First Clinical Retrospective Investigation of Limited Projection CBCT for Lung Tumor Localization in Patients Receiving SBRT Treatment. Phys Med Biol (2019) 64(10):10NT01–1. doi: 10.1088/1361-6560/ab1c0c PMC6635917 31018195

[118] Qin A Gersten D Liang J Liu Q Grill I Guerrero T. A Clinical 3D/4D CBCT-Based Treatment Dose Monitoring System. J Appl Clin Med Phys (2018) 19(6):166–76. doi: 10.1002/acm2.12474 PMC6236849 30306710

[119] Thengumpallil S Smith K Monnin P Bourhis J Bochud F Moeckli R. Difference in Performance Between 3D and 4D CBCT for Lung Imaging: A Dose and Image Quality Analysis. J Appl Clin Med Phys (2016) 17(6):97–106. doi: 10.1120/jacmp.v17i6.6459 27929485 PMC5690502

[120] den Otter LA Chen K Janssens G Meijers A Both S Langendijk JA. Technical Note: 4D Cone-Beam CT Reconstruction From Sparse-View CBCT Data for Daily Motion Assessment in Pencil Beam Scanned Proton Therapy (PBS-Pt). Med Phys (2020) 47(12):6381–7. doi: 10.1002/mp.14521 PMC7821169 33011990

[121] Kurz C Maspero M Savenije MHF Landry G Kamp F Pinto M. CBCT Correction Using a Cycle-Consistent Generative Adversarial Network and Unpaired Training to Enable Photon and Proton Dose Calculation. Phys Med Biol (2019) 64(22):225004. doi: 10.1088/1361-6560/ab4d8c 31610527

[122] Bryce-Atkinson A Marchant T Rodgers J Budgell G McWilliam A Faivre-Finn C. Quantitative Evaluation of 4D Cone Beam CT Scans With Reduced Scan Time in Lung Cancer Patients. Radiother Oncol (2019) 136:64–70. doi: 10.1016/j.radonc.2019.03.027 31015131 PMC6598855

[123] Shieh CC Gonzalez Y Li B Jia X Rit S Mory C. SPARE: Sparse-View Reconstruction Challenge for 4D Cone-Beam CT From a 1-Min Scan. Med Phys (2019) 46(9):3799–811. doi: 10.1002/mp.13687 PMC6739166 31247134

[124] Motion Management: 2-Axis and 4-Dimensional Cone Beam Ct. Hitachi Ltd (2021). Available at: https://www.hitachi.com/businesses/healthcare/products-support/pbt/probeat/motion/index.html.

[125] Schneider U Pedroni E. Proton Radiography as a Tool for Quality Control in Proton Therapy. Med Phys (1995) 22(4):353–63. doi: 10.1118/1.597470 7609715

[126] Schreuder AN Shamblin J. Proton Therapy Delivery: What is Needed in the Next Ten Years? Br J Radiol (2019) 93(1107):20190359. doi: 10.1259/bjr.20190359 31692372 PMC7066946

[127] DeJongh EA DeJongh DF Polnyi I Rykalin V Sarosiek C Coutrakon G. Technical Note: A Fast and Monolithic Prototype Clinical Proton Radiography System Optimized for Pencil Beam Scanning. Med Phys (2021) 48(3):1356–64. doi: 10.1002/mp.14700 PMC7965348 33382453

[128] Sarosiek C DeJongh EA Coutrakon G DeJongh DF Duffin KL Karonis NT. Analysis of Characteristics of Images Acquired With a Prototype Clinical Proton Radiography System. Med Phys (2021) 48(5):2271–8. doi: 10.1002/mp.14801 PMC8141022 33621368

[129] Farace P Righetto R Meijers A. Pencil Beam Proton Radiography Using a Multilayer Ionization Chamber. Phys Med Biol (2016) 61(11):4078–87. doi: 10.1088/0031-9155/61/11/4078 27164479

[130] Meijers A Seller OC Free J Bondesson D Seller Oria C Rabe M. Assessment of Range Uncertainty in Lung-Like Tissue Using a Porcine Lung Phantom and Proton Radiography. Phys Med Biol (2020) 65(15):155014. doi: 10.1088/1361-6560/ab91db 32392543

[131] Han B Xu XG Chen GT. Proton Radiography and Fluoroscopy of Lung Tumors: A Monte Carlo Study Using Patient-Specific 4DCT Phantoms. Med Phys (2011) 38(4):1903–11. doi: 10.1118/1.3555039 PMC3069996 21626923

[132] Gianoli C Göppel M Meyer S Palaniappan P Rädler M Kamp F. Patient-Specific CT Calibration Based on Ion Radiography for Different Detector Configurations in (1)H, (4)He and (12)C Ion Pencil Beam Scanning. Phys Med Biol (2020) 65(24):245014. doi: 10.1088/1361-6560/aba319 32629442

[133] Gehrke T Amato C Berke S Martišíková M. Theoretical and Experimental Comparison of Proton and Helium-Beam Radiography Using Silicon Pixel Detectors. Phys Med Biol (2018) 63(3):035037. doi: 10.1088/1361-6560/aaa60f 29311417

[134] Kopp B Meyer S Gianoli C Magallanes L Voss B Brons S. Experimental Comparison of Clinically Used Ion Beams for Imaging Applications Using a Range Telescope. Phys Med Biol (2020) 65(15):155004. doi: 10.1088/1361-6560/ab87f6 32268309

[135] Paganelli C Whelan B Peroni M Summers P Fast M van de Lindt T. MRI-Guidance for Motion Management in External Beam Radiotherapy: Current Status and Future Challenges. Phys Med Biol (2018) 63(22):22tr03. doi: 10.1088/1361-6560/aaebcf 30457121

[136] Stemkens B Paulson ES Tijssen RHN. Nuts and Bolts of 4D-MRI for Radiotherapy. Phys Med Biol (2018) 63(21):21tr01. doi: 10.1088/1361-6560/aae56d 30272573

[137] Moteabbed M Smeets J Hong TS Janssens G Labarbe R Wolfgang JA. Toward MR-Integrated Proton Therapy: Modeling the Potential Benefits for Liver Tumors. Phys Med Biol (2021) 66(19). doi: 10.1088/1361-6560/ac1ef2 34407528

[138] Fuchs H Padilla-Cabal F Zimmermann L Palmans H Georg D. MR-Guided Proton Therapy: Impact of Magnetic Fields on the Detector Response. Med Phys (2021) 48(5):2572–9. doi: 10.1002/mp.14660 PMC8251909 33326614

[139] Schellhammer SM Hoffmann AL Gantz S Smeets J van der Kraaij E Quets S. Integrating a Low-Field Open MR Scanner With a Static Proton Research Beam Line: Proof of Concept. Phys Med Biol (2018) 63(23):23lt01. doi: 10.1088/1361-6560/aaece8 30465549

[140] Liu R Lei Y Wang T Zhou J Roper J Lin L. Synthetic Dual-Energy CT for MRI-Only Based Proton Therapy Treatment Planning Using Label-GAN. Phys Med Biol (2021) 66(6):065014. doi: 10.1088/1361-6560/abe736 33596558 PMC11738296

[141] Freeman T. One Step Closer to Real-Time MR Imaging in Proton Therapy. Phys World (2021).

[142] Knopf A-C Lomax A. *In Vivo*proton Range Verification: A Review. Phys Med Biol (2013) 58(15):R131–R60. doi: 10.1088/0031-9155/58/15/R131 23863203

[143] Surti S Zou W Daube-Witherspoon ME McDonough J Karp JS. Design Study of an *in Situ* PET Scanner for Use in Proton Beam Therapy. Phys Med Biol (2011) 56(9):2667–85. doi: 10.1088/0031-9155/56/9/002 PMC3144472 21464528

[144] Zhu X El Fakhri G. Proton Therapy Verification With PET Imaging. Theranostics (2013) 3(10):731–40. doi: 10.7150/thno.5162 PMC3840408 24312147

[145] Kuess P Helmbrecht S Fiedler F Birkfellner W Enghardt W Hopfgartner J. Automated Evaluation of Setup Errors in Carbon Ion Therapy Using PET: Feasibility Study. Med Phys (2013) 40(12):121718. doi: 10.1118/1.4829595 24320504

[146] Parodi K Bortfeld T. A Filtering Approach Based on Gaussian-Powerlaw Convolutions for Local PET Verification of Proton Radiotherapy. Phys Med Biol (2006) 51(8):1991–2009. doi: 10.1088/0031-9155/51/8/003 16585841

[147] Ferrero V Fiorina E Morrocchi M Pennazio F Baroni G Battistoni G. Online Proton Therapy Monitoring: Clinical Test of a Silicon-Photodetector-Based in-Beam PET. Sci Rep (2018) 8(1):4100. doi: 10.1038/s41598-018-22325-6 29511282 PMC5840345

[148] Richter C Pausch G Barczyk S Priegnitz M Keitz I Thiele J. First Clinical Application of a Prompt Gamma Based *In Vivo* Proton Range Verification System. Radiother Oncol (2016) 118(2):232–7. doi: 10.1016/j.radonc.2016.01.004 26774764

[149] Xie Y Bentefour EH Janssens G Smeets J Vander Stappen F Hotoiu L. Prompt Gamma Imaging for In Vivo Range Verification of Pencil Beam Scanning Proton Therapy. Int J Radiat Oncol Biol Phys (2017) 99(1):210–8. doi: 10.1016/j.ijrobp.2017.04.027 28816148

[150] Tian L Huang Z Janssens G Landry G Dedes G Kamp F. Accounting for Prompt Gamma Emission and Detection for Range Verification in Proton Therapy Treatment Planning. Phys Med Biol (2021) 66(5):055005. doi: 10.1088/1361-6560/abc939 33171445

[151] Tian L Landry G Dedes G Pinto M Kamp F Belka C. A New Treatment Planning Approach Accounting for Prompt Gamma Range Verification and Interfractional Anatomical Changes. Phys Med Biol (2020) 65(9):095005. doi: 10.1088/1361-6560/ab7d15 32135530

[152] Muraro S Battistoni G Collamati F De Lucia E Faccini R Ferroni F. Monitoring of Hadrontherapy Treatments by Means of Charged Particle Detection. Front Oncol (2016) 6(177). doi: 10.3389/fonc.2016.00177 PMC4972018 27536555

[153] Rucinski A Rinaldi I De Lucia E Battistoni G Collamati F Faccini R. Internal Motion Tracking Based on Charged Secondary Proton Emission (Poster). 4D Treat Plann Workshop (2014).

[154] Bey A Ma J Furutani KM Herman MG Johnson JE Foote RL. Nuclear Fragmentation Imaging for Carbon-Ion Radiation Therapy Monitoring: An In Silico Study. Int J Part Ther (2021). doi: 10.14338/IJPT-20-00040.1 PMC9009459 35530183

[155] Rucinski A Battistoni G Collamati F De Lucia E Faccini R Frallicciardi PM. Secondary Radiation Measurements for Particle Therapy Applications: Charged Particles Produced By4he And12c Ion Beams in a PMMA Target at Large Angle. Phys Med Biol (2018) 63(5):055018. doi: 10.1088/1361-6560/aaa36a 29265011

[156] Félix-Bautista R Gehrke T Ghesquière-Diérickx L Reimold M Amato C Turecek D. Experimental Verification of a non-Invasive Method to Monitor the Lateral Pencil Beam Position in an Anthropomorphic Phantom for Carbon-Ion Radiotherapy. Phys Med Biol (2019) 64(17):175019. doi: 10.1088/1361-6560/ab2ca3 31239428

[157] Fischetti M Baroni G Battistoni G Bisogni G Cerello P Ciocca M. Inter-Fractional Monitoring of C12 Ions Treatments: Results From a Clinical Trial at the CNAO Facility. Sci Rep (2020) 10(1):20735. doi: 10.1038/s41598-020-77843-z 33244102 PMC7693236

[158] Nie W Jones KC Petro S Kassaee A Sehgal CM Avery S. Proton Range Verification in Homogeneous Materials Through Acoustic Measurements. Phys Med Biol (2018) 63(2):025036. doi: 10.1088/1361-6560/aa9c1f 29160776 PMC5845813

[159] Freijo C Herraiz JL Sanchez-Parcerisa D Udias JM. Dictionary-Based Protoacoustic Dose Map Imaging for Proton Range Verification. Photoacoustics (2021) 21:100240. doi: 10.1016/j.pacs.2021.100240 33520652 PMC7820918

[160] Yu Y Li Z Zhang D Xing L Peng H. Simulation Studies of Time Reversal-Based Protoacoustic Reconstruction for Range and Dose Verification in Proton Therapy. Med Phys (2019) 46(8):3649–62. doi: 10.1002/mp.13661 31199511

[161] Jones KC Seghal CM Avery S. How Proton Pulse Characteristics Influence Protoacoustic Determination of Proton-Beam Range: Simulation Studies. Phys Med Biol (2016) 61(6):2213–42. doi: 10.1088/0031-9155/61/6/2213 26913839

[162] Rietzel E Bert C. Respiratory Motion Management in Particle Therapy. Med Phys (2010) 37(2):449–60. doi: 10.1118/1.3250856 20229853

[163] Knopf A Nill S Yohannes I Graeff C Dowdell S Kurz C. Challenges of Radiotherapy: Report on the 4D Treatment Planning Workshop 2013. Physica Med (2014) 30(7):809–15. doi: 10.1016/j.ejmp.2014.07.341 25172392

[164] Riboldi M Orecchia R Baroni G. Real-Time Tumour Tracking in Particle Therapy: Technological Developments and Future Perspectives. Lancet Oncol (2012) 13(9):e383–e91. doi: 10.1016/S1470-2045(12)70243-7 22935238

[165] Eley JG Newhauser WD Richter D Lüchtenborg R Saito N Bert C. Robustness of Target Dose Coverage to Motion Uncertainties for Scanned Carbon Ion Beam Tracking Therapy of Moving Tumors. Phys Med Biol (2015) 60(4):1717–40. doi: 10.1088/0031-9155/60/4/1717 PMC4384336 25650520

[166] van de Water S Kreuger R Zenklusen S Hug E Lomax AJ. Tumour Tracking With Scanned Proton Beams: Assessing the Accuracy and Practicalities. Phys Med Biol (2009) 54(21):6549–63. doi: 10.1088/0031-9155/54/21/007 19826204

[167] Krieger M Giger A Jud C Duetschler A Salomir R Bieri O. Liver-Ultrasound-Guided Lung Tumour Tracking for Scanned Proton Therapy: A Feasibility Study. Phys Med Biol (2021) 66(3):035011. doi: 10.1088/1361-6560/abcde6 33238246

[168] Prall M Kaderka R Saito N Graeff C Bert C Durante M. Ion Beam Tracking Using Ultrasound Motion Detection. Med Phys (2014) 41(4):041708. doi: 10.1118/1.4868459 24694128

[169] Lis M Newhauser W Donetti M Wolf M Steinsberger T Paz A. A Modular System for Treating Moving Anatomical Targets With Scanned Ion Beams at Multiple Facilities: Pre-Clinical Testing for Quality and Safety of Beam Delivery. Front Oncol (2021) 11(323). doi: 10.3389/fonc.2021.620388 PMC8018284 33816251

[170] Mazzucconi D Agosteo S Ferrarini M Fontana L Lante V Pullia M. Mixed Particle Beam for Simultaneous Treatment and Online Range Verification in Carbon Ion Therapy: Proof-of-Concept Study. Med Phys (2018) 45(11):5234–43. doi: 10.1002/mp.13219 30269349

[171] Graeff C Weber U Schuy C Saito N Volz L Piersimoni P. [OA027] Helium as a Range Probe in Carbon Ion Therapy. Physica Med (2018) 52:11. doi: 10.1016/j.ejmp.2018.06.099

[172] Volz L Kelleter L Brons S Burigo L Graeff C Niebuhr NI. Experimental Exploration of a Mixed Helium/Carbon Beam for Online Treatment Monitoring in Carbon Ion Beam Therapy. Phys Med Biol (2020) 65(5):055002. doi: 10.1088/1361-6560/ab6e52 31962302

[173] Durante M Parodi K. Radioactive Beams in Particle Therapy: Past, Present, and Future. Front Phys (2020) 8:326. doi: 10.3389/fphy.2020.00326 PMC7116396 33224941

[174] Yang J Chu D Dong L Court LE. Advantages of Simulating Thoracic Cancer Patients in an Upright Position. Pract Radiat Oncol (2014) 4(1):e53–e8. doi: 10.1016/j.prro.2013.04.005 24621432

[175] Durante M Debus J Loeffler JS. Physics and Biomedical Challenges of Cancer Therapy With Accelerated Heavy Ions. Nat Rev Phys (2021) 3(12):777–90. doi: 10.1038/s42254-021-00368-5 PMC7612063 34870097

[176] Wilson JD Hammond EM Higgins GS Petersson K. Ultra-High Dose Rate (FLASH) Radiotherapy: Silver Bullet or Fool's Gold? Front Oncol (2020) 9:1563. doi: 10.3389/fonc.2019.01563 PMC6979639 32010633

[177] Montay-Gruel P Petersson K Jaccard M Boivin G Germond JF Petit B. Irradiation in a Flash: Unique Sparing of Memory in Mice After Whole Brain Irradiation With Dose Rates Above 100Gy/s. Radiother Oncol (2017) 124(3):365–9. doi: 10.1016/j.radonc.2017.05.003 28545957

[178] Vozenin MC De Fornel P Petersson K Favaudon V Jaccard M Germond JF. The Advantage of FLASH Radiotherapy Confirmed in Mini-Pig and Cat-Cancer Patients. Clin Cancer Res (2019) 25(1):35–42. doi: 10.1158/1078-0432.CCR-17-3375 29875213

[179] Vozenin MC Hendry JH Limoli CL. Biological Benefits of Ultra-High Dose Rate FLASH Radiotherapy: Sleeping Beauty Awoken. Clin Oncol (R Coll Radiol) (2019) 31(7):407–15. doi: 10.1016/j.clon.2019.04.001 PMC6850216 31010708

[180] Esplen N Mendonca MS Bazalova-Carter M. Physics and Biology of Ultrahigh Dose-Rate (FLASH) Radiotherapy: A Topical Review. Phys Med Biol (2020) 65(23):23tr03. doi: 10.1088/1361-6560/abaa28 32721941

[181] Diffenderfer ES Verginadis II Kim MM Shoniyozov K Velalopoulou A Goia D. Design, Implementation, and in Vivo Validation of a Novel Proton FLASH Radiation Therapy System. Int J Radiat Oncol Biol Phys (2020) 106(2):440–8. doi: 10.1016/j.ijrobp.2019.10.049 PMC7325740 31928642

[182] Patriarca A Fouillade C Auger M Martin F Pouzoulet F Nauraye C. Experimental Set-Up for FLASH Proton Irradiation of Small Animals Using a Clinical System. Int J Radiat Oncol Biol Phys (2018) 102(3):619–26. doi: 10.1016/j.ijrobp.2018.06.403 30017793

[183] Zou W Diffenderfer ES Cengel KA Kim MM Avery S Konzer J. Current Delivery Limitations of Proton PBS for FLASH. Radiother Oncol (2021) 155:212–8. doi: 10.1016/j.radonc.2020.11.002 33186682

[184] Jolly S Owen H Schippers M Welsch C. Technical Challenges for FLASH Proton Therapy. Physica Med (2020) 78:71–82. doi: 10.1016/j.ejmp.2020.08.005 32947086

[185] van de Water S Safai S Schippers JM Weber DC Lomax AJ. Towards FLASH Proton Therapy: The Impact of Treatment Planning and Machine Characteristics on Achievable Dose Rates. Acta Oncol (2019) 58(10):1463–9. doi: 10.1080/0284186X.2019.1627416 31241377

[186] Teoh M Clark CH Wood K Whitaker S Nisbet A. Volumetric Modulated Arc Therapy: A Review of Current Literature and Clinical Use in Practice. Br J Radiol (2011) 84(1007):967–96. doi: 10.1259/bjr/22373346 PMC3473700 22011829

[187] IBA Initiates Global DynamicARC Consortium for the Roll-Out of Proton Arc Therapy [Press Release]. (2021).

[188] Chang S Liu G Zhao L Dilworth JT Zheng W Jawad S. Feasibility Study: Spot-Scanning Proton Arc Therapy (SPArc) for Left-Sided Whole Breast Radiotherapy. Radiat Oncol (2020) 15(1):232. doi: 10.1186/s13014-020-01676-3 33028378 PMC7542109

[189] Ding X Li X Zhang JM Kabolizadeh P Stevens C Yan D. Spot-Scanning Proton Arc (SPArc) Therapy: The First Robust and Delivery-Efficient Spot-Scanning Proton Arc Therapy. Int J Radiat Oncol Biol Phys (2016) 96(5):1107–16. doi: 10.1016/j.ijrobp.2016.08.049 27869083

[190] Gu W Ruan D Lyu Q Zou W Dong L Sheng K. A Novel Energy Layer Optimization Framework for Spot-Scanning Proton Arc Therapy. Med Phys (2020) 47(5):2072–84. doi: 10.1002/mp.14083 PMC7234928 32040214

[191] Mein S Tessonnier T Kopp B Harrabi S Abdollahi A Debus J. Spot-Scanning Hadron Arc (SHArc) Therapy: A Study With Light and Heavy Ions. Adv Radiat Oncol (2021) 6(3):100661. doi: 10.1016/j.adro.2021.100661 33817410 PMC8010580

[192] Carabe-Fernandez A Bertolet-Reina A Karagounis I Huynh K Dale RG. Is There a Role for Arcing Techniques in Proton Therapy? Br J Radiol (2020) 93(1107):20190469. doi: 10.1259/bjr.20190469 31860338 PMC7066964

[193] Seco J Gu G Marcelos T Kooy H Willers H. Proton Arc Reduces Range Uncertainty Effects and Improves Conformality Compared With Photon Volumetric Modulated Arc Therapy in Stereotactic Body Radiation Therapy for non-Small Cell Lung Cancer. Int J Radiat Oncol Biol Phys (2013) 87(1):188–94. doi: 10.1016/j.ijrobp.2013.04.048 23920395

[194] Toussaint L Indelicato DJ Holgersen KS Petersen JBB Stokkevåg CH Lassen-Ramshad Y. Towards Proton Arc Therapy: Physical and Biologically Equivalent Doses With Increasing Number of Beams in Pediatric Brain Irradiation. Acta Oncol (2019) 58(10):1451–6. doi: 10.1080/0284186X.2019.1639823 31303090

[195] Sejnowski TJ. The Deep Learning Revolution. Cambridge, MA: The MIT Press (2018).

[196] LeCun Y Bengio Y Hinton G. Deep Learning. Nature (2015) 521(7553):436–44. doi: 10.1038/nature14539 26017442

[197] El Naqa I Haider MA Giger ML Ten Haken RK. Artificial Intelligence: Reshaping the Practice of Radiological Sciences in the 21st Century. Br J Radiol (2020) 93(1106):20190855–. doi: 10.1259/bjr.20190855 PMC7055429 31965813

[198] van der Heyden B Cohilis M Souris K de Freitas Nascimento L Sterpin E. Artificial Intelligence Supported Single Detector Multi-Energy Proton Radiography System. Phys Med Biol (2021) 66(10):105001. doi: 10.1088/1361-6560/abe918 33621962

[199] Lalonde A Winey B Verburg J Paganetti H Sharp GC. Evaluation of CBCT Scatter Correction Using Deep Convolutional Neural Networks for Head and Neck Adaptive Proton Therapy. Phys Med Biol (2020) 65(24):245022. doi: 10.1088/1361-6560/ab9fcb PMC8920050 32580174

[200] Thummerer A Seller Oria C Zaffino P Meijers A Guterres Marmitt G Wijsman R. Clinical Suitability of Deep Learning Based Synthetic CTs for Adaptive Proton Therapy of Lung Cancer. Med Phys (2021) 48(12):7673–84. doi: 10.1002/mp.15333 PMC9299115 34725829

[201] Harms J Lei Y Wang T McDonald M Ghavidel B Stokes W. Cone-Beam CT-Derived Relative Stopping Power Map Generation *via* Deep Learning for Proton Radiotherapy. Med Phys (2020) 47(9):4416–27. doi: 10.1002/mp.14347 PMC11650372 32579710

[202] Elmahdy MS Jagt T Zinkstok RT Qiao Y Shahzad R Sokooti H. Robust Contour Propagation Using Deep Learning and Image Registration for Online Adaptive Proton Therapy of Prostate Cancer. Med Phys (2019) 46(8):3329–43. doi: 10.1002/mp.13620 PMC6852565 31111962

[203] Hirai R Sakata Y Tanizawa A Mori S. Real-Time Tumor Tracking Using Fluoroscopic Imaging With Deep Neural Network Analysis. Physica Med (2019) 59:22–9. doi: 10.1016/j.ejmp.2019.02.006 30928062

[204] Mylonas A Keall PJ Booth JT Shieh C-C Eade T Poulsen PR. A Deep Learning Framework for Automatic Detection of Arbitrarily Shaped Fiducial Markers in Intrafraction Fluoroscopic Images. Med Phys (2019) 46(5):2286–97. doi: 10.1002/mp.13519 30929254

[205] Zhao W Shen L Han B Yang Y Cheng K Toesca DAS. Markerless Pancreatic Tumor Target Localization Enabled By Deep Learning. Int J Radiat Oncol Biol Phys (2019) 105(2):432–9. doi: 10.1016/j.ijrobp.2019.05.071 PMC6732032 31201892

[206] Kim KH Park K Kim H Jo B Ahn SH Kim C. Facial Expression Monitoring System for Predicting Patient's Sudden Movement During Radiotherapy Using Deep Learning. J Appl Clin Med Phys (2020) 21(8):191–9. doi: 10.1002/acm2.12945 PMC7484824 32515552

[207] Lin H Zou W Li T Feigenberg SJ Teo B-KK Dong L. A Super-Learner Model for Tumor Motion Prediction and Management in Radiation Therapy: Development and Feasibility Evaluation. Sci Rep (2019) 9(1):14868. doi: 10.1038/s41598-019-51338-y 31619736 PMC6795883

[208] Cui S Tseng H-H Pakela J Ten Haken RK El Naqa I. Introduction to Machine and Deep Learning for Medical Physicists. Med Phys (2020) 47(5):e127–e47. doi: 10.1002/mp.14140 PMC7331753 32418339

[209] Caruana R Lou Y Gehrke J Koch P Sturm M Elhadad N. Intelligible Models for HealthCare: Predicting Pneumonia Risk and Hospital 30-Day Readmission. In: Proceedings of the 21th ACM SIGKDD International Conference on Knowledge Discovery and Data Mining Sydney, Australia:Association for Computing Machinery (2015).

[210] Luo Y Tseng H-H Cui S Wei L Ten Haken RK El Naqa I. Balancing Accuracy and Interpretability of Machine Learning Approaches for Radiation Treatment Outcomes Modeling. BJR Open (2019) 1(1):20190021. doi: 10.1259/bjro.20190021 PMC7592485 33178948

[211] Philbrick KA Yoshida K Inoue D Akkus Z Kline TL Weston AD. What Does Deep Learning See? Insights From a Classifier Trained to Predict Contrast Enhancement Phase From CT Images. AJR Am J Roentgenol (2018) 211(6):1184–93. doi: 10.2214/AJR.18.20331 30403527

[212] Seah JCY Tang JSN Kitchen A Gaillard F Dixon AF. Chest Radiographs in Congestive Heart Failure: Visualizing Neural Network Learning. Radiology (2019) 290(2):514–22. doi: 10.1148/radiol.2018180887 30398431

